# Supplementary material for: Induced Pluripotent Stem Cell-Derived Cardiomyocytes Therapy for Ischemic Heart Disease in Animal Model: A Meta-Analysis
Source: Int J Mol Sci. 2024 Jan 12;25(2):987. doi: 10.3390/ijms25020987 (PMC10815661; doi:10.3390/ijms25020987)
Supplement: Supplementary file 1 [file ijms-25-00987-s001.zip › ijms-2789206-supplementary.pdf]

Induced pluripotent Stem Cell-derived cardiomyocytes therapy for ischemic heart disease in animal model: Systematic Review and Meta-analysis

Search query: ("iPScells" OR "induced pluripotent stem cells" OR "pluripotent stem cells" OR "reprogrammed stem cells") AND ("Ischemic Heart Disease" OR "coronary artery disease" OR "myocardial infarction" OR "heart ischemia" OR “agina” OR “arteriosclerosis coronary” OR “Myocardial Reperfusion”)

Study or Subgroup	iPSC-CM Mean	iPSC-CM SD	iPSC-CM Total	Control Mean	Control SD	Control Total	Weight	Mean Difference IV, Random, 95% CI
**6.1.1 Small animal**								
A. A. Winters, et al. 2016	43.7	7.8	8	29.7	8.8	6	0.7%	14.00 [5.12, 22.88]
A. A. Winters, et al. 2016	45.6	6.5	17	32.5	6.4	15	1.1%	13.10 [8.62, 17.58]
A.Chow, et al. 2017	55.3	8	7	47.3	6.1	7	0.9%	8.00 [0.55, 15.45]
A.Chow, et al. 2017	50.6	8.9	9	44.5	7.8	8	0.8%	6.10 [-1.84, 14.04]
A.Tachibana, et al 2017	28.4	9.2	14	24.1	6	10	1.0%	4.30 [-1.79, 10.39]
A.Tachibana, et al 2017	36.6	5.8	14	19.9	5.7	10	1.1%	16.70 [12.04, 21.36]
C.Fan, et als 2019	15.3	1.4	12	8.9	1.4	12	1.4%	6.40 [5.28, 7.52]
C.Fan, et als 2020	25.8	0.4	10	13.6	0.6	10	1.4%	12.20 [11.75, 12.65]
D. Biagi, et als 2021	46.3	3.4	7	35.2	2.9	6	1.2%	11.10 [7.68, 14.52]
F.Zhang, et als 2020	61.77	9.06	6	38.84	16.78	6	0.4%	22.93 [7.67, 38.19]
F.Zhang, et als 2020	62.9	2.6	6	46.1	17.1	6	0.4%	16.80 [2.96, 30.64]
H.Li, et al 2021	34.6	3	5	20.8	1.5	5	1.3%	13.80 [10.86, 16.74]
H.Li, et al 2021	27.8	3.1	5	17.2	1.9	5	1.3%	10.60 [7.41, 13.79]
H.Li, et al 2021	31.1	2.4	5	24.2	2.8	5	1.3%	6.90 [3.67, 10.13]
H.Li, et al 2023	25.9	2	5	17.5	2.2	5	1.3%	8.40 [5.79, 11.01]
J.Li, et al 2017	50.63	6.41	12	37.5	5.15	17	1.2%	13.13 [8.75, 17.51]
J.Stępniewski, et al 2020	30.57	3.02	7	25.14	6.84	11	1.1%	5.43 [0.81, 10.05]
J.Stępniewski, et al 2020	39.02	6.64	7	21.32	7.65	11	0.9%	17.70 [11.02, 24.38]
J.Stępniewski, et al 2020	18.51	3.21	7	23.94	3.42	12	1.3%	-5.43 [-8.50, -2.36]
J.Stępniewski, et al 2020	30.78	3.82	7	23.53	3.42	12	1.2%	7.25 [3.82, 10.68]
J.Yokoyama, et al 2021	50.6	3.7	10	44.8	3.4	10	1.3%	5.80 [2.69, 8.91]
J.Yokoyama, et al 2021	53.7	3.3	10	41	2.3	10	1.3%	12.70 [10.21, 15.19]
K.Miki, et al 2012	48.6	4.7	15	36.9	5	15	1.2%	11.70 [8.23, 15.17]
K.Neef, et al 2021	50	3.3	8	43.1	1.2	9	1.3%	6.90 [4.48, 9.32]
K.Neef, et al 2021	49.5	1.2	8	44	2.5	9	1.4%	5.50 [3.67, 7.33]
K.Neef, et al 2021	51.8	2.8	8	44.2	2.6	9	1.3%	7.60 [5.02, 10.18]
K.Neef, et al 2021	46.3	4	8	44.1	2.3	9	1.3%	2.20 [-0.95, 5.35]
KPMM.Ja, et al 2016	36.53	5.84	4	32.97	18.35	8	0.4%	3.56 [-10.38, 17.50]
KPMM.Ja, et al 2016	25.69	11.35	4	32.97	18.35	8	0.3%	-7.28 [-24.17, 9.61]
L.Citro, et al 2014	81.1	10.5	5	46.9	8.8	5	0.5%	34.20 [22.19, 46.21]
L.Saludas, et al 2019	41.25	2.57	8	33.21	1.86	9	1.3%	8.04 [5.88, 10.20]
L.Saludas, et al 2019	32.7	2.4	11	29.5	1.1	13	1.4%	3.20 [1.66, 4.74]
L.Zhang, et al 2016	43.5	2.1	7	32.5	3.2	7	1.3%	11.00 [8.16, 13.84]
L.Zhang, et al 2016	41.4	2.6	7	39.9	5.1	7	1.2%	1.50 [-2.74, 5.74]
O.I.García, et al 2015	35.5	4.7	8	36	4.3	8	1.1%	-0.50 [-4.91, 3.91]
O.I.García, et al 2015	34.7	5.5	8	33.2	2.3	8	1.2%	1.50 [-2.63, 5.63]
O.I.García, et al 2015	32.6	3.9	8	28.4	8.2	8	1.0%	4.20 [-2.09, 10.49]
R.Ma, et al 2018	46.43	8.4	16	28.3	1.5	8	1.2%	18.13 [13.88, 22.38]
S.G.Ong, et al 2015	20.8	1.3	8	19.2	1.5	8	1.4%	1.60 [0.22, 2.98]
S.G.Ong, et al 2015	24.4	1.1	8	14.5	1.8	8	1.4%	9.90 [8.44, 11.36]
S.J.Sun, et al 2021	46.3	5.8	8	38	5.5	8	1.0%	8.30 [2.76, 13.84]
S.Mattapally, et al 2018	55.4	5	6	28.3	1.3	6	1.2%	27.10 [22.97, 31.23]
S.V.Rojas, et al 2016	34	4	21	19	2	10	1.3%	15.00 [12.89, 17.11]
S.V.Rojas, et al 2016	47	3	28	39	5	15	1.3%	8.00 [5.24, 10.76]
T.Higuchi, et al 2015	51.09	0.81	5	50.46	0.75	6	1.4%	0.63 [-0.30, 1.56]
T.Higuchi, et al 2015	54	0.7	6	48.1	1.2	6	1.4%	5.90 [4.79, 7.01]
T.Higuchi, et al 2015	53.28	1.2	6	49.52	1	6	1.4%	3.76 [2.51, 5.01]
T.Higuchi, et al 2015	54.6	1	6	48.2	1.1	6	1.4%	6.40 [5.21, 7.59]
T.Higuchi, et al 2015	54.9	0.7	6	48.2	1.3	6	1.4%	6.70 [5.52, 7.88]
T.Samura, et al 2020	44.7	2.5	10	35.8	0.6	10	1.4%	8.90 [7.31, 10.49]
T.Samura, et al 2020	46.7	2.3	10	36.1	0.6	10	1.4%	10.60 [9.13, 12.07]
T.Samura, et al 2020	48.1	5.9	10	34.8	2.5	10	1.2%	13.30 [9.33, 17.27]
T.Samura, et al 2020	42.4	2.5	10	35.8	0.7	10	1.4%	6.60 [4.99, 8.21]
W.Bian, et al 2021	27.6	2.7	6	18.9	3.8	6	1.2%	8.70 [4.97, 12.43]
W.H.Lee, et al 2017	54	3.3	8	45.4	2.8	7	1.3%	8.60 [5.51, 11.69]
W.Zhu, et al 2018	42.2	3.6	9	32.3	2	9	1.3%	9.90 [7.21, 12.59]
W.Zhu, et al 2018	38.4	4.3	10	36.9	2.5	10	1.3%	1.50 [-1.58, 4.58]
X.Guan, et al 2020	52.41	3	9	41.67	5.1	8	1.2%	10.74 [6.70, 14.78]
X.Jiang, et al 2020	50.3	7.6	9	45.91	7	9	0.9%	4.39 [-2.36, 11.14]
X.Jiang, et al 2020	54.7	6.42	9	43.22	7.71	8	0.9%	11.48 [4.69, 18.27]
X.Jiang, et al 2020	48.3	8.1	9	46.73	8.1	9	0.9%	1.57 [-5.91, 9.05]
X.Lou, et al 2019	38.6	5.3	9	19.7	2.2	7	1.2%	18.90 [15.07, 22.73]
X.Lou, et al 2019	30.7	2.6	9	27.2	3.9	7	1.2%	3.50 [0.15, 6.85]
X.Qu, et al 2023	45.3	2.3	7	32.3	5.6	7	1.1%	13.00 [8.52, 17.48]
X.Qu, et al 2023	45.91	3.93	7	32.3	1.9	7	1.3%	13.61 [10.38, 16.84]
X.Qu, et al 2023	43.14	3.48	7	36.3	8.5	7	0.9%	6.84 [0.04, 13.64]
X.Qu, et al 2023	41.84	1.56	7	34.4	5.6	7	1.2%	7.44 [3.13, 11.75]
X.Sun, et al 2020	31.5	5	8	24.5	2.7	4	1.2%	7.00 [2.64, 11.36]
Y.Gao, et al 2021	57.3	6.8	5	33.4	8.8	5	0.7%	23.90 [14.15, 33.65]
Y.Gao, et al 2021	53	6.5	5	42	9.5	5	0.6%	11.00 [0.91, 21.09]
Y.H.Fang, et al 2020	56.2	1.1	6	48.6	2	6	1.4%	7.60 [5.77, 9.43]
Y.H.Fang, et al 2020	65.6	1.1	6	51	0.7	6	1.4%	14.60 [13.56, 15.64]
Y.Jiang, et al 2021	56.35	6.18	0	43.22	6.5	9		Not estimable
Y.Jiang, et al 2023	39.47	1.75	13	37.85	1.89	13	1.4%	1.62 [0.22, 3.02]
Y.Jiang, et al 2023	39.8	1.2	13	33.9	1.5	13	1.4%	5.90 [4.86, 6.94]
Y.Jiang, et al 2023	39.19	1.61	6	36.17	1.4	6	1.4%	3.02 [1.31, 4.73]
Y.Jiang, et al 2023	39.61	1.26	6	36.45	1.54	6	1.4%	3.16 [1.57, 4.75]
Z.Tao, et al 2021	46.35	10.3	11	42.49	3.86	9	0.9%	3.86 [-2.73, 10.45]
Z.Tao, et al 2021	49.36	10.3	11	45.92	7.73	9	0.8%	3.44 [-4.47, 11.35]
**Subtotal (95% CI)**			**676**			**663**	**89.6%**	**8.33 [7.18, 9.47]**
Heterogeneity: Tau² = 21.50; Chi² = 1602.79, df = 77 (P < 0.00001); I² = 95%								
Test for overall effect: Z = 14.26 (P < 0.00001)								
**6.1.2 Large animal**								
H.m.Li 2023	53.47	7.8	14	54.86	12.08	3	0.4%	-1.39 [-15.66, 12.88]
H.m.Li 2023	50.99	8.99	14	49.79	4.91	3	0.9%	1.20 [-6.08, 8.48]
H.m.Li 2023	45.36	7.76	14	42.82	2.54	3	1.1%	2.54 [-2.44, 7.52]
H.m.Li 2023	51.28	4.6	11	45.77	3.38	3	1.1%	5.51 [0.82, 10.20]
H.m.Li 2023	49.79	7.07	11	42.39	14.36	3	0.3%	7.40 [-9.38, 24.18]
M.Ishida, et al 2019	45.5	2	7	30.2	1.4	8	1.4%	15.30 [13.53, 17.07]
M.Kawamura, et al 2012	53.2	4.3	6	38.3	4.3	6	1.1%	14.90 [10.03, 19.77]
M.Kawamura, et al 2012	51.6	4.9	6	36	5.9	6	1.0%	15.60 [9.46, 21.74]
M.Zhao, et al 2021	44.98	2.1	7	42.15	2.77	7	1.3%	2.83 [0.25, 5.41]
Y.Shiba, et al 2016	57.44	7.3	5	45.94	5.06	5	0.8%	11.50 [3.71, 19.29]
Y.Shiba, et al 2016	60.08	4.21	5	48.88	4.98	5	1.0%	11.20 [5.48, 16.92]
**Subtotal (95% CI)**			**100**			**52**	**10.4%**	**8.38 [4.03, 12.72]**
Heterogeneity: Tau² = 41.61; Chi² = 91.43, df = 10 (P < 0.00001); I² = 89%								
Test for overall effect: Z = 3.78 (P = 0.0002)								
**Total (95% CI)**			**776**			**715**	**100.0%**	**8.35 [7.26, 9.44]**
Heterogeneity: Tau² = 21.94; Chi² = 1708.13, df = 88 (P < 0.00001); I² = 95%								
Test for overall effect: Z = 14.99 (P < 0.00001)								
Test for subgroup differences: Chi² = 0.00, df = 1 (P = 0.98), I² = 0%								

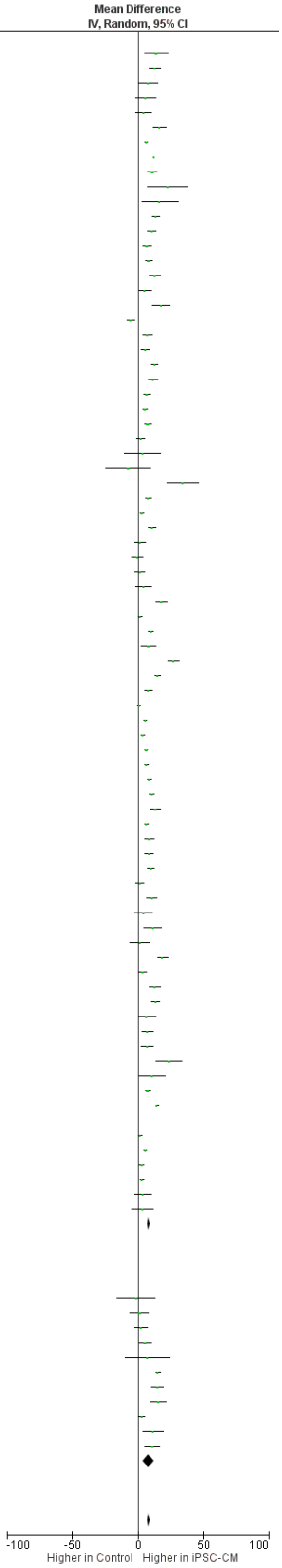

Figure S1. Cell-based effect on animal ejection fraction by animal size

	iPSC-MC			Control				Mean Difference
**Study or Subgroup**	**Mean**	**SD**	**Total**	**Mean**	**SD**	**Total**	**Weight**	**IV, Random, 95% CI**
**7.1.1 < 4 weeks**								
A.Chow, et al. 2017	50.6	8.9	9	44.5	7.8	8	0.8%	6.10 [-1.84, 14.04]
A.Tachibana, et al 2017	28.4	9.2	14	24.1	6	10	1.0%	4.30 [-1.79, 10.39]
F.Zhang, et als 2020	62.9	2.6	6	46.1	17.1	6	0.4%	16.80 [2.96, 30.64]
H.Li, et al 2021	31.1	2.4	5	24.2	2.8	5	1.3%	6.90 [3.67, 10.13]
H.Li, et al 2021	34.6	3	5	20.8	1.5	5	1.3%	13.80 [10.86, 16.74]
H.m.Li 2023	53.47	7.8	14	54.86	12.08	3	0.4%	-1.39 [-15.66, 12.88]
H.m.Li 2023	50.99	8.99	14	49.79	4.91	3	0.9%	1.20 [-6.08, 8.48]
J.Stępniewski, et al 2020	18.51	3.21	7	23.94	3.42	12	1.3%	-5.43 [-8.50, -2.36]
J.Stępniewski, et al 2020	30.78	3.82	7	23.53	3.42	12	1.2%	7.25 [3.82, 10.68]
J.Yokoyama, et al 2021	50.6	3.7	10	44.8	3.4	10	1.3%	5.80 [2.69, 8.91]
K.Neef, et al 2021	46.3	4	8	44.1	2.3	9	1.3%	2.20 [-0.95, 5.35]
K.Neef, et al 2021	50	3.3	8	43.1	1.2	9	1.3%	6.90 [4.48, 9.32]
K.Neef, et al 2021	49.5	1.2	8	44	2.5	9	1.4%	5.50 [3.67, 7.33]
KPMM.Ja, et al 2016	36.68	2.04	4	32.97	18.35	8	0.5%	3.71 [-9.16, 16.58]
L.Saludas, et al 2019	32.7	2.4	11	29.5	1.1	13	1.4%	3.20 [1.66, 4.74]
L.Zhang, et al 2016	41.4	2.6	7	39.9	5.1	7	1.2%	1.50 [-2.74, 5.74]
O.I.García, et al 2015	35.5	4.7	8	36	4.3	8	1.1%	-0.50 [-4.91, 3.91]
S.G.Ong, et al 2015	20.8	1.3	8	19.2	1.5	8	1.4%	1.60 [0.22, 2.98]
S.V.Rojas, et al 2016	47	3	28	39	5	15	1.3%	8.00 [5.24, 10.76]
T.Higuchi, et al 2015	53.28	1.2	6	49.52	1	6	1.4%	3.76 [2.51, 5.01]
T.Higuchi, et al 2015	51.09	0.81	5	50.46	0.75	6	1.4%	0.63 [-0.30, 1.56]
T.Higuchi, et al 2015	54.6	1	6	48.2	1.1	6	1.4%	6.40 [5.21, 7.59]
T.Higuchi, et al 2015	54	0.7	6	48.1	1.2	6	1.4%	5.90 [4.79, 7.01]
T.Higuchi, et al 2015	54.9	0.7	6	48.2	1.3	6	1.4%	6.70 [5.52, 7.88]
T.Samura, et al 2020	42.4	2.5	10	35.8	0.7	10	1.4%	6.60 [4.99, 8.21]
T.Samura, et al 2020	44.7	2.5	10	35.8	0.6	10	1.4%	8.90 [7.31, 10.49]
T.Samura, et al 2020	46.7	2.3	10	36.1	0.6	10	1.4%	10.60 [9.13, 12.07]
W.Zhu, et al 2018	38.4	4.3	10	36.9	2.5	10	1.3%	1.50 [-1.58, 4.58]
X.Jiang, et al 2020	48.3	8.1	9	46.73	8.1	9	0.8%	1.57 [-5.91, 9.05]
X.Jiang, et al 2020	50.3	7.6	9	45.9	7	9	0.9%	4.40 [-2.35, 11.15]
X.Lou, et al 2019	30.7	2.6	9	27.2	3.9	7	1.2%	3.50 [0.15, 6.85]
X.Qu, et al 2023	45.3	2.3	7	32.3	5.6	7	1.1%	13.00 [8.52, 17.48]
X.Qu, et al 2023	45.91	3.93	7	32.3	1.9	7	1.3%	13.61 [10.38, 16.84]
X.Qu, et al 2023	41.84	1.56	7	34.4	5.6	7	1.2%	7.44 [3.13, 11.75]
Y.Gao, et al 2021	53	6.5	5	42	9.5	5	0.6%	11.00 [0.91, 21.09]
Y.H.Fang, et al 2020	56.2	1.1	6	48.6	2	6	1.4%	7.60 [5.77, 9.43]
Y.Jiang, et al 2023	39.47	1.75	13	37.85	1.89	13	1.4%	1.62 [0.22, 3.02]
Z.Tao, et al 2021	49.36	10.3	11	45.92	7.73	9	0.8%	3.44 [-4.47, 11.35]
**Subtotal (95% CI)**			**333**			**309**	**43.7%**	**5.38 [4.13, 6.63]**
Heterogeneity: $Tau^2 = 11.36$; $Chi^2 = 400.02$, df = 37 (P < 0.00001); $I^2 = 91\%$								
Test for overall effect: Z = 8.42 (P < 0.00001)								
**7.1.5 4 - 8 weeks**								
A. A. Winters, et al. 2016	39.1	6.1	8	29.7	8.8	6	0.8%	9.40 [1.19, 17.61]
A.Tachibana, et al 2017	36.6	5.8	14	19.9	5.7	10	1.1%	16.70 [12.04, 21.36]
C.Fan, et als 2020	25.8	0.4	10	13.6	0.6	10	1.4%	12.20 [11.75, 12.65]
D. Biagi, et als 2021	46.3	3.4	7	35.2	2.9	6	1.2%	11.10 [7.68, 14.52]
F.Zhang, et als 2020	61.77	9.06	6	38.84	16.78	6	0.4%	22.93 [7.67, 38.19]
H.Li, et al 2021	27.8	3.1	5	17.2	1.9	5	1.3%	10.60 [7.41, 13.79]
H.Li, et al 2023	25.9	2	5	17.5	2.2	5	1.3%	8.40 [5.79, 11.01]
H.m.Li 2023	45.36	7.76	14	42.82	2.54	3	1.1%	2.54 [-2.44, 7.52]
J.Li, et al 2017	50.63	6.41	12	37.5	5.15	17	1.2%	13.13 [8.75, 17.51]
J.Stępniewski, et al 2020	30.57	3.02	7	25.14	6.84	11	1.1%	5.43 [0.81, 10.05]
J.Stępniewski, et al 2020	39.02	6.64	7	21.32	7.65	11	0.9%	17.70 [11.02, 24.38]
J.Yokoyama, et al 2021	53.7	3.3	10	41	2.3	10	1.3%	12.70 [10.21, 15.19]
K.Miki, et al 2012	48.6	4.7	15	36.9	5	15	1.2%	11.70 [8.23, 15.17]
K.Neef, et al 2021	51.8	2.8	8	44.2	2.6	9	1.3%	7.60 [5.02, 10.18]
KPMM.Ja, et al 2016	25.69	11.35	4	32.97	18.35	8	0.3%	-7.28 [-24.17, 9.61]
L.Citro, et al 2014	81.1	10.5	5	46.9	8.8	5	0.5%	34.20 [22.19, 46.21]
L.Zhang, et al 2016	43.5	2.1	7	32.5	3.2	7	1.3%	11.00 [8.16, 13.84]
M.Kawamura, et al 2012	53.2	4.3	6	38.3	4.3	6	1.1%	14.90 [10.03, 19.77]
M.Zhao, et al 2021	44.98	2.1	7	42.15	2.77	7	1.3%	2.83 [0.25, 5.41]
O.I.García, et al 2015	34.7	5.5	8	33.2	2.3	8	1.2%	1.50 [-2.63, 5.63]
R.Ma, et al 2018	46.43	8.4	16	28.3	1.5	8	1.2%	18.13 [13.88, 22.38]
S.G.Ong, et al 2015	24.4	1.1	8	14.5	1.8	8	1.4%	9.90 [8.44, 11.36]
S.J.Sun, et al 2021	46.3	5.8	8	38	5.5	8	1.0%	8.30 [2.76, 13.84]
S.Mattapally, et al 2018	55.4	5	6	28.3	1.3	6	1.2%	27.10 [22.97, 31.23]
S.V.Rojas, et al 2016	34	4	21	19	2	10	1.3%	15.00 [12.89, 17.11]
T.Samura, et al 2020	48.1	5.9	10	34.8	2.5	10	1.2%	13.30 [9.33, 17.27]
W.Bian, et al 2021	27.6	2.7	6	18.9	3.8	6	1.2%	8.70 [4.97, 12.43]
W.H.Lee, et al 2017	54	3.3	8	45.4	2.8	7	1.3%	8.60 [5.51, 11.69]
W.Zhu, et al 2018	42.2	3.6	9	32.3	2	9	1.3%	9.90 [7.21, 12.59]
X.Guan, et al 2020	52.41	3	9	41.67	5.1	8	1.2%	10.74 [6.70, 14.78]
X.Jiang, et al 2020	54.7	6.42	9	43.22	7.71	8	0.9%	11.48 [4.69, 18.27]
X.Lou, et al 2019	38.6	5.3	9	19.7	2.2	7	1.2%	18.90 [15.07, 22.73]
X.Qu, et al 2023	43.14	3.48	7	36.3	8.5	7	0.9%	6.84 [0.04, 13.64]
X.Sun, et al 2020	31.5	5	8	24.5	2.7	4	1.2%	7.00 [2.64, 11.36]
Y.Gao, et al 2021	57.3	6.8	5	33.4	8.8	5	0.7%	23.90 [14.15, 33.65]
Y.H.Fang, et al 2020	65.6	1.1	6	51	0.7	6	1.4%	14.60 [13.56, 15.64]
Y.Jiang, et al 2021	56.35	6.18	0	43.22	6.5	9		Not estimable
Y.Jiang, et al 2023	39.8	1.2	13	33.9	1.5	13	1.4%	5.90 [4.86, 6.94]
Y.Shiba, et al 2016	57.44	7.3	5	45.94	5.06	5	0.8%	11.50 [3.71, 19.29]
Z.Tao, et al 2021	46.35	10.3	11	42.49	3.86	9	0.9%	3.86 [-2.73, 10.45]
**Subtotal (95% CI)**			**339**			**318**	**43.0%**	**11.23 [9.84, 12.61]**
Heterogeneity: $Tau^2 = 13.95$; $Chi^2 = 405.57$, df = 38 (P < 0.00001); $I^2 = 91\%$								
Test for overall effect: Z = 15.86 (P < 0.00001)								
**7.1.9 >= 8 weeks**								
A. A. Winters, et al. 2016	33.4	6.7	17	32.5	6.4	15	1.1%	0.90 [-3.64, 5.44]
A.Chow, et al. 2017	55.3	8	7	47.3	6.1	7	0.9%	8.00 [0.55, 15.45]
C.Fan, et als 2019	15.3	1.4	12	8.9	1.4	12	1.4%	6.40 [5.28, 7.52]
H.m.Li 2023	51.28	4.6	11	45.77	3.38	3	1.1%	5.51 [0.82, 10.20]
H.m.Li 2023	49.79	7.07	11	42.39	14.36	3	0.3%	7.40 [-9.38, 24.18]
L.Saludas, et al 2019	41.25	2.57	8	33.21	1.86	9	1.3%	8.04 [5.88, 10.20]
M.Ishida, et al 2019	45.5	2	7	30.2	1.4	8	1.4%	15.30 [13.53, 17.07]
M.Kawamura, et al 2012	51.6	4.9	6	36	5.9	6	1.0%	15.60 [9.46, 21.74]
O.I.García, et al 2015	32.6	3.9	8	28.4	8.2	8	1.0%	4.20 [-2.09, 10.49]
Y.Jiang, et al 2023	39.61	1.26	6	36.45	1.54	6	1.4%	3.16 [1.57, 4.75]
Y.Jiang, et al 2023	39.19	1.61	6	36.17	1.4	6	1.4%	3.02 [1.31, 4.73]
Y.Shiba, et al 2016	60.08	4.21	5	48.88	4.98	5	1.0%	11.20 [5.48, 16.92]
**Subtotal (95% CI)**			**104**			**88**	**13.2%**	**7.26 [4.39, 10.13]**
Heterogeneity: $Tau^2 = 19.68$; $Chi^2 = 146.86$, df = 11 (P < 0.00001); $I^2 = 93\%$								
Test for overall effect: Z = 4.96 (P < 0.00001)								
**Total (95% CI)**			**776**			**715**	**100.0%**	**8.18 [7.09, 9.27]**
Heterogeneity: $Tau^2 = 21.99$; $Chi^2 = 1711.73$, df = 88 (P < 0.00001); $I^2 = 95\%$								
Test for overall effect: Z = 14.67 (P < 0.00001)								
Test for subgroup differences: $Chi^2 = 37.86$, df = 2 (P < 0.00001), $I^2 = 94.7\%$								

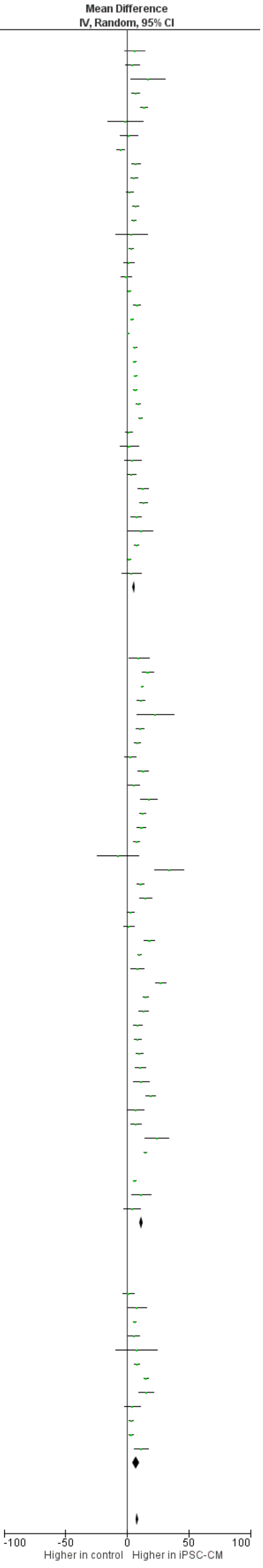

Figure S2. Cell-based effect on animal ejection fraction by follow-up time

Study or Subgroup	iPSC-CM Mean	iPSC-CM SD	iPSC-CM Total	Control Mean	Control SD	Control Total	Weight	Mean Difference IV, Random, 95% CI
**8.1.1 Intramyocardial**								
A. A. Winters, et al. 2016	43.7	7.8	8	29.7	8.8	6	0.6%	14.00 [5.12, 22.88]
A. A. Winters, et al. 2016	45.6	6.5	17	32.5	6.4	15	1.0%	13.10 [8.62, 17.58]
A.Chow, et al. 2017	55.3	8	7	47.3	6.1	7	0.7%	8.00 [0.55, 15.45]
A.Chow, et al. 2017	50.6	8.9	9	44.5	7.8	8	0.7%	6.10 [-1.84, 14.04]
A.Tachibana, et al 2017	28.4	9.2	14	24.1	6	10	0.9%	4.30 [-1.79, 10.39]
A.Tachibana, et al 2017	36.6	5.8	14	19.9	5.7	10	1.0%	16.70 [12.04, 21.36]
C.Fan, et als 2019	15.3	1.4	12	8.9	1.4	12	1.2%	6.40 [5.28, 7.52]
D. Biagi, et als 2021	46.3	3.4	7	35.2	2.9	6	1.1%	11.10 [7.68, 14.52]
F.Zhang, et als 2020	61.77	9.06	6	38.84	16.78	6	0.3%	22.93 [7.67, 38.19]
F.Zhang, et als 2020	62.9	2.6	6	46.1	17.1	6	0.4%	16.80 [2.96, 30.64]
H.Li, et al 2021	34.6	3	5	20.8	1.5	5	1.1%	13.80 [10.86, 16.74]
H.Li, et al 2021	27.8	3.1	5	17.2	1.9	5	1.1%	10.60 [7.41, 13.79]
H.Li, et al 2021	31.1	2.4	5	24.2	2.8	5	1.1%	6.90 [3.67, 10.13]
H.Li, et al 2023	25.9	2	5	17.5	2.2	5	1.1%	8.40 [5.79, 11.01]
H.m.Li 2023	53.47	9.39	6	54.86	12.08	3	0.3%	-1.39 [-16.99, 14.21]
H.m.Li 2023	53.88	9.79	6	49.79	4.91	3	0.6%	4.09 [-5.51, 13.69]
H.m.Li 2023	50.41	8.73	6	42.82	2.54	3	0.7%	7.59 [0.04, 15.14]
H.m.Li 2023	51.84	5.3	6	45.77	3.38	3	0.9%	6.07 [0.36, 11.78]
H.m.Li 2023	46.73	8.58	6	42.39	14.36	3	0.3%	4.34 [-13.30, 21.98]
J.Stępniewski, et al 2020	18.51	3.21	7	23.94	3.42	12	1.1%	-5.43 [-8.50, -2.36]
J.Stępniewski, et al 2020	30.78	3.82	7	23.53	3.42	12	1.1%	7.25 [3.82, 10.68]
J.Stępniewski, et al 2020	30.57	3.02	7	25.14	6.84	11	1.0%	5.43 [0.81, 10.05]
J.Stępniewski, et al 2020	39.02	6.64	7	21.32	7.65	11	0.8%	17.70 [11.02, 24.38]
K.Neef, et al 2021	50	3.3	8	43.1	1.2	9	1.2%	6.90 [4.48, 9.32]
K.Neef, et al 2021	49.5	1.2	8	44	2.5	9	1.2%	5.50 [3.67, 7.33]
K.Neef, et al 2021	51.8	2.8	8	44.2	2.6	9	1.1%	7.60 [5.02, 10.18]
K.Neef, et al 2021	46.3	4	8	44.1	2.3	9	1.1%	2.20 [-0.95, 5.35]
KPMM.Ja, et al 2016	36.53	5.84	4	32.97	18.35	8	0.4%	3.56 [-10.38, 17.50]
KPMM.Ja, et al 2016	25.69	11.35	4	32.97	18.35	8	0.3%	-7.28 [-24.17, 9.61]
L.Citro, et al 2014	81.1	10.5	5	46.9	8.8	5	0.5%	34.20 [22.19, 46.21]
L.Citro, et al 2014	81.1	10.5	5	46.9	8.8	5	0.5%	34.20 [22.19, 46.21]
L.Saludas, et al 2019	41.25	2.57	8	33.21	1.86	9	1.2%	8.04 [5.88, 10.20]
L.Saludas, et al 2019	32.7	2.4	11	29.5	1.1	13	1.2%	3.20 [1.66, 4.74]
M.Zhao, et al 2021	44.98	2.1	7	42.15	2.77	7	1.1%	2.83 [0.25, 5.41]
O.I.García, et al 2015	34.7	5.5	8	33.2	2.3	8	1.0%	1.50 [-2.63, 5.63]
O.I.García, et al 2015	32.6	3.9	8	28.4	8.2	8	0.8%	4.20 [-2.09, 10.49]
O.I.García, et al 2015	35.5	4.7	8	36	4.3	8	1.0%	-0.50 [-4.91, 3.91]
R.Ma, et al 2018	46.43	8.4	16	28.3	1.5	8	1.0%	18.13 [13.88, 22.38]
S.G.Ong, et al 2015	20.8	1.3	8	19.2	1.5	8	1.2%	1.60 [0.22, 2.98]
S.G.Ong, et al 2015	24.4	1.1	8	14.5	1.8	8	1.2%	9.90 [8.44, 11.36]
S.J.Sun, et al 2021	46.3	5.8	8	38	5.5	8	0.9%	8.30 [2.76, 13.84]
S.J.Sun, et al 2021	46.3	5.8	8	38	5.5	8	0.9%	8.30 [2.76, 13.84]
S.V.Rojas, et al 2016	34	4	21	19	2	10	1.2%	15.00 [12.89, 17.11]
S.V.Rojas, et al 2016	47	3	28	39	5	15	1.1%	8.00 [5.24, 10.76]
W.Bian, et al 2021	27.6	2.7	6	18.9	3.8	6	1.1%	8.70 [4.97, 12.43]
W.H.Lee, et al 2017	54	3.3	8	45.4	2.8	7	1.1%	8.60 [5.51, 11.69]
W.Zhu, et al 2018	42.2	3.6	9	32.3	2	9	1.1%	9.90 [7.21, 12.59]
W.Zhu, et al 2018	38.4	4.3	10	36.9	2.5	10	1.1%	1.50 [-1.58, 4.58]
X.Guan, et al 2020	52.41	3	9	41.67	5.1	8	1.0%	10.74 [6.70, 14.78]
X.Jiang, et al 2020	50.3	7.6	9	45.91	7	9	0.8%	4.39 [-2.36, 11.14]
X.Jiang, et al 2020	54.7	6.42	9	43.22	7.71	8	0.8%	11.48 [4.69, 18.27]
X.Jiang, et al 2020	48.3	8.1	9	46.73	8.1	9	0.7%	1.57 [-5.91, 9.05]
X.Lou, et al 2019	38.6	5.3	9	19.7	2.2	7	1.1%	18.90 [15.07, 22.73]
X.Lou, et al 2019	30.7	2.6	9	27.2	3.9	7	1.1%	3.50 [0.15, 6.85]
X.Sun, et al 2020	31.5	5	8	24.5	2.7	4	1.0%	7.00 [2.64, 11.36]
Y.Gao, et al 2021	57.3	6.8	5	33.4	8.8	5	0.6%	23.90 [14.15, 33.65]
Y.Gao, et al 2021	53	6.5	5	42	9.5	5	0.6%	11.00 [0.91, 21.09]
Y.H.Fang, et al 2020	56.2	1.1	6	48.6	2	6	1.2%	7.60 [5.77, 9.43]
Y.H.Fang, et al 2020	65.6	1.1	6	51	0.7	6	1.2%	14.60 [13.56, 15.64]
Y.Jiang, et al 2021	52.26	5.65	9	43.22	6.5	9	0.9%	9.04 [3.41, 14.67]
Y.Jiang, et al 2023	39.47	1.75	13	37.85	1.89	13	1.2%	1.62 [0.22, 3.02]
Y.Jiang, et al 2023	39.8	1.2	13	33.9	1.5	13	1.2%	5.90 [4.86, 6.94]
Y.Jiang, et al 2023	39.19	1.61	6	36.17	1.4	6	1.2%	3.02 [1.31, 4.73]
Y.Jiang, et al 2023	39.61	1.26	6	36.45	1.54	6	1.2%	3.16 [1.57, 4.75]
Y.Shiba, et al 2016	57.44	7.3	5	45.94	5.06	5	0.7%	11.50 [3.71, 19.29]
Y.Shiba, et al 2016	60.08	4.21	5	48.88	4.98	5	0.9%	11.20 [5.48, 16.92]
**Subtotal (95% CI)**			**549**			**510**	**61.3%**	**8.09 [6.79, 9.39]**
Heterogeneity: Tau² = 21.72; Chi² = 797.15, df = 65 (P < 0.00001); I² = 92%								
Test for overall effect: Z = 12.19 (P < 0.00001)								
**8.1.2 Intravenous**								
H.m.Li 2023	57.76	4.48	5	54.86	12.08	3	0.4%	2.90 [-11.32, 17.12]
H.m.Li 2023	52.65	7.15	5	49.79	4.91	3	0.7%	2.86 [-5.52, 11.24]
H.m.Li 2023	42.04	4.08	5	42.82	2.54	3	1.0%	-0.78 [-5.37, 3.81]
H.m.Li 2023	50.96	4.08	5	45.77	3.38	3	0.9%	5.19 [-0.05, 10.43]
H.m.Li 2023	53.47	1.43	5	42.39	14.36	3	0.3%	11.08 [-5.22, 27.38]
**Subtotal (95% CI)**			**25**			**15**	**3.3%**	**2.34 [-0.74, 5.43]**
Heterogeneity: Tau² = 0.15; Chi² = 4.04, df = 4 (P = 0.40); I² = 1%								
Test for overall effect: Z = 1.49 (P = 0.14)								
**8.1.3 Intracoronary**								
H.m.Li 2023	46.33	4.08	3	54.86	12.08	3	0.4%	-8.53 [-22.96, 5.90]
H.m.Li 2023	42.45	6.53	3	49.79	4.91	3	0.6%	-7.34 [-16.59, 1.91]
H.m.Li 2023	40.82	6.94	3	42.82	2.54	3	0.7%	-2.00 [-10.36, 6.36]
**Subtotal (95% CI)**			**9**			**9**	**1.6%**	**-5.05 [-10.74, 0.65]**
Heterogeneity: Tau² = 0.00; Chi² = 0.97, df = 2 (P = 0.62); I² = 0%								
Test for overall effect: Z = 1.74 (P = 0.08)								
**8.1.4 Bio-engineered tissue**								
C.Fan, et als 2020	25.8	0.4	10	13.6	0.6	10	1.2%	12.20 [11.75, 12.65]
J.Li, et al 2017	50.63	6.41	12	37.5	5.15	17	1.0%	13.13 [8.75, 17.51]
J.Yokoyama, et al 2021	50.6	3.7	10	44.8	3.4	10	1.1%	5.80 [2.69, 8.91]
J.Yokoyama, et al 2021	53.7	3.3	10	41	2.3	10	1.2%	12.70 [10.21, 15.19]
K.Miki, et al 2012	48.6	4.7	15	36.9	5	15	1.1%	11.70 [8.23, 15.17]
L.Zhang, et al 2016	43.5	2.1	7	32.5	3.2	7	1.1%	11.00 [8.16, 13.84]
L.Zhang, et al 2016	41.4	2.6	7	39.9	5.1	7	1.0%	1.50 [-2.74, 5.74]
M.Ishida, et al 2019	45.5	2	7	30.2	1.4	8	1.2%	15.30 [13.53, 17.07]
M.Kawamura, et al 2012	53.2	4.3	6	38.3	4.3	6	1.0%	14.90 [10.03, 19.77]
M.Kawamura, et al 2012	51.6	4.9	6	36	5.9	6	0.9%	15.60 [9.46, 21.74]
S.Mattapally, et al 2018	55.4	5	6	28.3	1.3	6	1.0%	27.10 [22.97, 31.23]
T.Higuchi, et al 2015	54.6	1	6	48.2	1.1	6	1.2%	6.40 [5.21, 7.59]
T.Higuchi, et al 2015	54.9	0.7	6	48.2	1.3	6	1.2%	6.70 [5.52, 7.88]
T.Higuchi, et al 2015	51.09	0.81	5	50.46	0.75	6	1.2%	0.63 [-0.30, 1.56]
T.Higuchi, et al 2015	54	0.7	6	48.1	1.2	6	1.2%	5.90 [4.79, 7.01]
T.Higuchi, et al 2015	53.28	1.2	6	49.52	1	6	1.2%	3.76 [2.51, 5.01]
T.Samura, et al 2020	44.7	2.5	10	35.8	0.6	10	1.2%	8.90 [7.31, 10.49]
T.Samura, et al 2020	46.7	2.3	10	36.1	0.6	10	1.2%	10.60 [9.13, 12.07]
T.Samura, et al 2020	48.1	5.9	10	34.8	2.5	10	1.0%	13.30 [9.33, 17.27]
T.Samura, et al 2020	42.4	2.5	10	35.8	0.7	10	1.2%	6.60 [4.99, 8.21]
X.Qu, et al 2023	45.91	3.93	7	32.3	1.9	7	1.1%	13.61 [10.38, 16.84]
X.Qu, et al 2023	43.14	3.48	7	36.3	8.5	7	0.8%	6.84 [0.04, 13.64]
X.Qu, et al 2023	41.84	1.56	7	34.4	5.6	7	1.0%	7.44 [3.13, 11.75]
X.Qu, et al 2023	45.3	2.3	7	32.3	5.6	7	1.0%	13.00 [8.52, 17.48]
Y.Jiang, et al 2021	60.45	3.39	9	43.22	6.5	9	1.0%	17.23 [12.44, 22.02]
Y.Jiang, et al 2023	39.47	1.75	13	37.85	1.89	13	1.2%	1.62 [0.22, 3.02]
Y.Jiang, et al 2023	39.8	1.2	13	33.9	1.5	13	1.2%	5.90 [4.86, 6.94]
Y.Jiang, et al 2023	39.19	1.61	6	36.17	1.4	6	1.2%	3.02 [1.31, 4.73]
Y.Jiang, et al 2023	39.61	1.26	6	36.45	1.54	6	1.2%	3.16 [1.57, 4.75]
Z.Tao, et al 2021	46.35	10.3	11	42.49	3.86	9	0.8%	3.86 [-2.73, 10.45]
Z.Tao, et al 2021	49.36	10.3	11	45.92	7.73	9	0.7%	3.44 [-4.47, 11.35]
**Subtotal (95% CI)**			**262**			**265**	**33.8%**	**9.00 [7.20, 10.80]**
Heterogeneity: Tau² = 23.15; Chi² = 1063.77, df = 30 (P < 0.00001); I² = 97%								
Test for overall effect: Z = 9.79 (P < 0.00001)								
**Total (95% CI)**			**845**			**799**	**100.0%**	**8.01 [6.99, 9.03]**
Heterogeneity: Tau² = 21.94; Chi² = 1932.64, df = 104 (P < 0.00001); I² = 95%								
Test for overall effect: Z = 15.37 (P < 0.00001)								
Test for subgroup differences: Chi² = 32.72, df = 3 (P < 0.00001), I² = 90.8%								

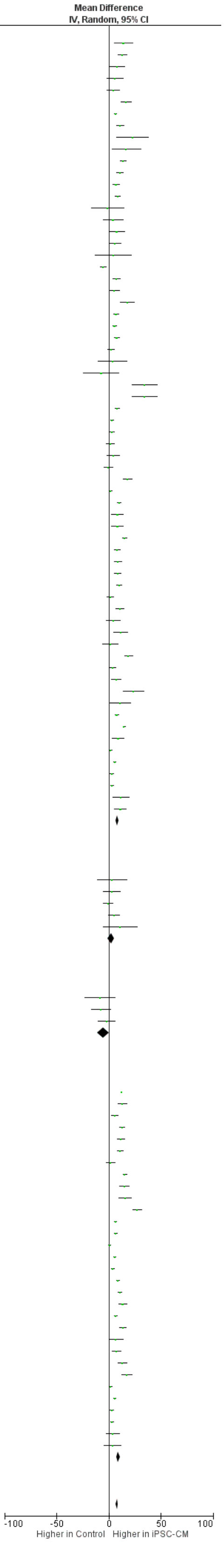

Figure S3. Cell-based effect on animal ejection fraction by delivery method

Study or Subgroup	iPSC-CM Mean	iPSC-CM SD	iPSC-CM Total	Control Mean	Control SD	Control Total	Weight	Mean Difference IV, Random, 95% CI
**9.1.1 acute (< 1week)**								
A. A. Winters, et al. 2016	39.1	6.1	8	29.7	8.8	6	0.8%	9.40 [1.19, 17.61]
A. A. Winters, et al. 2016	33.4	6.7	17	32.5	6.4	15	1.1%	0.90 [-3.64, 5.44]
A.Chow, et al. 2017	55.3	8	7	47.3	6.1	7	0.8%	8.00 [0.55, 15.45]
A.Chow, et al. 2017	50.6	8.9	9	44.5	7.8	8	0.8%	6.10 [-1.84, 14.04]
A.Tachibana, et al 2017	28.4	9.2	14	24.1	6	10	1.0%	4.30 [-1.79, 10.39]
A.Tachibana, et al 2017	36.6	5.8	14	19.9	5.7	10	1.1%	16.70 [12.04, 21.36]
C.Fan, et als 2019	15.3	1.4	12	8.9	1.4	12	1.4%	6.40 [5.28, 7.52]
C.Fan, et als 2020	25.8	0.4	10	13.6	0.6	10	1.4%	12.20 [11.75, 12.65]
F.Zhang, et als 2020	61.77	9.06	6	38.84	16.78	6	0.4%	22.93 [7.67, 38.19]
F.Zhang, et als 2020	62.9	2.6	6	46.1	17.1	6	0.4%	16.80 [2.96, 30.64]
H.Li, et al 2021	34.6	3	5	20.8	1.5	5	1.3%	13.80 [10.86, 16.74]
H.Li, et al 2021	27.8	3.1	5	17.2	1.9	5	1.2%	10.60 [7.41, 13.79]
H.Li, et al 2021	31.1	2.4	5	24.2	2.8	5	1.2%	6.90 [3.67, 10.13]
H.Li, et al 2023	25.9	2	5	17.5	2.2	5	1.3%	8.40 [5.79, 11.01]
H.m.Li 2023	53.47	7.8	14	54.86	12.08	3	0.4%	-1.39 [-15.66, 12.88]
H.m.Li 2023	50.99	8.99	14	49.79	4.91	3	0.9%	1.20 [-6.08, 8.48]
H.m.Li 2023	45.36	7.76	14	42.82	2.54	3	1.1%	2.54 [-2.44, 7.52]
H.m.Li 2023	51.28	4.6	11	45.77	3.38	3	1.1%	5.51 [0.82, 10.20]
H.m.Li 2023	49.79	7.07	11	42.39	14.36	3	0.3%	7.40 [-9.38, 24.18]
J.Li, et al 2017	50.63	6.41	12	37.5	5.15	17	1.1%	13.13 [8.75, 17.51]
J.Stępniewski, et al 2020	30.57	3.02	7	25.14	6.84	11	1.1%	5.43 [0.81, 10.05]
J.Stępniewski, et al 2020	39.02	6.64	7	21.32	7.65	11	0.9%	17.70 [11.02, 24.38]
J.Stępniewski, et al 2020	18.51	3.21	7	23.94	3.42	12	1.3%	-5.43 [-8.50, -2.36]
J.Stępniewski, et al 2020	30.78	3.82	7	23.53	3.42	12	1.2%	7.25 [3.82, 10.68]
K.Neef, et al 2021	50	3.3	8	43.1	1.2	9	1.3%	6.90 [4.48, 9.32]
K.Neef, et al 2021	49.5	1.2	8	44	2.5	9	1.3%	5.50 [3.67, 7.33]
K.Neef, et al 2021	51.8	2.8	8	44.2	2.6	9	1.3%	7.60 [5.02, 10.18]
K.Neef, et al 2021	46.3	4	8	44.1	2.3	9	1.3%	2.20 [-0.95, 5.35]
KPMM.Ja, et al 2016	36.68	2.04	4	32.97	18.35	8	0.5%	3.71 [-9.16, 16.58]
KPMM.Ja, et al 2016	25.69	11.35	4	32.97	18.35	8	0.3%	-7.28 [-24.17, 9.61]
L.Citro, et al 2014	81.1	10.5	5	46.9	8.8	5	0.5%	34.20 [22.19, 46.21]
L.Saludas, et al 2019	41.25	2.57	8	33.21	1.86	9	1.3%	8.04 [5.88, 10.20]
L.Saludas, et al 2019	32.7	2.4	11	29.5	1.1	13	1.4%	3.20 [1.66, 4.74]
L.Zhang, et al 2016	43.5	2.1	7	32.5	3.2	7	1.3%	11.00 [8.16, 13.84]
L.Zhang, et al 2016	41.4	2.6	7	39.9	5.1	7	1.2%	1.50 [-2.74, 5.74]
M.Zhao, et al 2021	44.98	2.1	7	42.15	2.77	7	1.3%	2.83 [0.25, 5.41]
O.I.García, et al 2015	34.7	5.5	8	33.2	2.3	8	1.2%	1.50 [-2.63, 5.63]
O.I.García, et al 2015	32.6	3.9	8	28.4	8.2	8	1.0%	4.20 [-2.09, 10.49]
O.I.García, et al 2015	35.5	4.7	8	36	4.3	8	1.1%	-0.50 [-4.91, 3.91]
R.Ma, et al 2018	46.43	8.4	16	28.3	1.5	8	1.2%	18.13 [13.88, 22.38]
S.G.Ong, et al 2015	20.8	1.3	8	19.2	1.5	8	1.4%	1.60 [0.22, 2.98]
S.G.Ong, et al 2015	24.4	1.1	8	14.5	1.8	8	1.4%	9.90 [8.44, 11.36]
S.Mattapally, et al 2018	55.4	5	6	28.3	1.3	6	1.2%	27.10 [22.97, 31.23]
S.V.Rojas, et al 2016	34	4	21	19	2	10	1.3%	15.00 [12.89, 17.11]
S.V.Rojas, et al 2016	47	3	28	39	5	15	1.3%	8.00 [5.24, 10.76]
T.Samura, et al 2020	44.7	2.5	10	35.8	0.6	10	1.4%	8.90 [7.31, 10.49]
T.Samura, et al 2020	46.7	2.3	10	36.1	0.6	10	1.4%	10.60 [9.13, 12.07]
T.Samura, et al 2020	48.1	5.9	10	34.8	2.5	10	1.2%	13.30 [9.33, 17.27]
T.Samura, et al 2020	42.4	2.5	10	35.8	0.7	10	1.4%	6.60 [4.99, 8.21]
W.Bian, et al 2021	27.6	2.7	6	18.9	3.8	6	1.2%	8.70 [4.97, 12.43]
W.H.Lee, et al 2017	54	3.3	8	45.4	2.8	7	1.3%	8.60 [5.51, 11.69]
W.Zhu, et al 2018	42.2	3.6	9	32.3	2	9	1.3%	9.90 [7.21, 12.59]
W.Zhu, et al 2018	38.4	4.3	10	36.9	2.5	10	1.3%	1.50 [-1.58, 4.58]
X.Jiang, et al 2020	50.4	7.6	9	45.9	7	9	0.9%	4.50 [-2.25, 11.25]
X.Jiang, et al 2020	54.7	6.42	9	43.22	7.71	8	0.9%	11.48 [4.69, 18.27]
X.Jiang, et al 2020	48.3	8.1	9	46.73	8.1	9	0.8%	1.57 [-5.91, 9.05]
X.Lou, et al 2019	38.6	5.3	9	19.7	2.2	7	1.2%	18.90 [15.07, 22.73]
X.Lou, et al 2019	30.7	2.6	9	27.2	3.9	7	1.2%	3.50 [0.15, 6.85]
Y.Gao, et al 2021	57.3	6.8	5	33.4	8.8	5	0.7%	23.90 [14.15, 33.65]
Y.Gao, et al 2021	53	6.5	5	42	9.5	5	0.6%	11.00 [0.91, 21.09]
Y.H.Fang, et al 2020	56.2	1.1	6	48.6	2	6	1.3%	7.60 [5.77, 9.43]
Y.H.Fang, et al 2020	65.6	1.1	6	51	0.7	6	1.4%	14.60 [13.56, 15.64]
Y.Jiang, et al 2021	56.36	6.18	18	43.22	6.5	9	1.1%	13.14 [8.02, 18.26]
Z.Tao, et al 2021	46.35	10.3	11	42.49	3.86	9	0.9%	3.86 [-2.73, 10.45]
Z.Tao, et al 2021	49.36	10.3	11	45.92	7.73	9	0.8%	3.44 [-4.47, 11.35]
**Subtotal (95% CI)**			**603**			**528**	**69.7%**	**8.21 [6.93, 9.49]**
Heterogeneity: Tau^2 = 21.08; Chi^2 = 981.01, df = 64 (P < 0.00001); I^2 = 93%								
Test for overall effect: Z = 12.58 (P < 0.00001)								
**9.1.3 Sub-acute (1 week - 1 month)**								
D. Biagi, et als 2021	46.3	3.4	7	35.2	2.9	6	1.2%	11.10 [7.68, 14.52]
J.Yokoyama, et al 2021	50.6	3.7	10	44.8	3.4	10	1.3%	5.80 [2.69, 8.91]
J.Yokoyama, et al 2021	53.7	3.3	10	41	2.3	10	1.3%	12.70 [10.21, 15.19]
K.Miki, et al 2012	48.6	4.7	15	36.9	5	15	1.2%	11.70 [8.23, 15.17]
S.J.Sun, et al 2021	46.3	5.8	8	38	5.5	8	1.0%	8.30 [2.76, 13.84]
T.Higuchi, et al 2015	51.09	0.81	5	50.46	0.75	6	1.4%	0.63 [-0.30, 1.56]
T.Higuchi, et al 2015	54	0.7	6	48.1	1.2	6	1.4%	5.90 [4.79, 7.01]
T.Higuchi, et al 2015	53.28	1.2	6	49.52	1	6	1.4%	3.76 [2.51, 5.01]
T.Higuchi, et al 2015	54.6	1	6	48.2	1.1	6	1.4%	6.40 [5.21, 7.59]
T.Higuchi, et al 2015	54.9	0.7	6	48.2	1.3	6	1.4%	6.70 [5.52, 7.88]
X.Guan, et al 2020	52.41	3	9	41.67	5.1	8	1.2%	10.74 [6.70, 14.78]
X.Qu, et al 2023	45.3	2.3	7	32.3	5.6	7	1.1%	13.00 [8.52, 17.48]
X.Qu, et al 2023	45.91	3.93	7	32.3	1.9	7	1.2%	13.61 [10.38, 16.84]
X.Qu, et al 2023	43.14	3.48	7	36.3	8.5	7	0.9%	6.84 [0.04, 13.64]
X.Qu, et al 2023	41.84	1.56	7	34.4	5.6	7	1.1%	7.44 [3.13, 11.75]
X.Sun, et al 2020	31.5	5	8	24.5	2.7	4	1.1%	7.00 [2.64, 11.36]
Y.Jiang, et al 2023	39.47	1.75	13	37.85	1.89	13	1.4%	1.62 [0.22, 3.02]
Y.Jiang, et al 2023	39.8	1.2	13	33.9	1.5	13	1.4%	5.90 [4.86, 6.94]
Y.Jiang, et al 2023	39.19	1.61	6	36.17	1.4	6	1.4%	3.02 [1.31, 4.73]
Y.Jiang, et al 2023	39.61	1.26	6	36.45	1.54	6	1.4%	3.16 [1.57, 4.75]
Y.Shiba, et al 2016	57.44	7.3	5	45.94	5.06	5	0.8%	11.50 [3.71, 19.29]
Y.Shiba, et al 2016	60.08	4.21	5	48.88	4.98	5	1.0%	11.20 [5.48, 16.92]
**Subtotal (95% CI)**			**172**			**167**	**26.9%**	**7.15 [5.66, 8.63]**
Heterogeneity: Tau^2 = 9.85; Chi^2 = 261.30, df = 21 (P < 0.00001); I^2 = 92%								
Test for overall effect: Z = 9.44 (P < 0.00001)								
**9.1.4 Chronic (> 1 month)**								
M.Ishida, et al 2019	45.5	2	7	30.2	1.4	8	1.3%	15.30 [13.53, 17.07]
M.Kawamura, et al 2012	53.2	4.3	6	38.3	4.3	6	1.1%	14.90 [10.03, 19.77]
M.Kawamura, et al 2012	51.6	4.9	6	36	5.9	6	1.0%	15.60 [9.46, 21.74]
**Subtotal (95% CI)**			**19**			**20**	**3.4%**	**15.28 [13.67, 16.88]**
Heterogeneity: Tau^2 = 0.00; Chi^2 = 0.03, df = 2 (P = 0.98); I^2 = 0%								
Test for overall effect: Z = 18.64 (P < 0.00001)								
**Total (95% CI)**			**794**			**715**	**100.0%**	**8.23 [7.15, 9.32]**
Heterogeneity: Tau^2 = 21.98; Chi^2 = 1715.32, df = 89 (P < 0.00001); I^2 = 95%								
Test for overall effect: Z = 14.85 (P < 0.00001)								
Test for subgroup differences: Chi^2 = 62.85, df = 2 (P < 0.00001), I^2 = 96.8%								

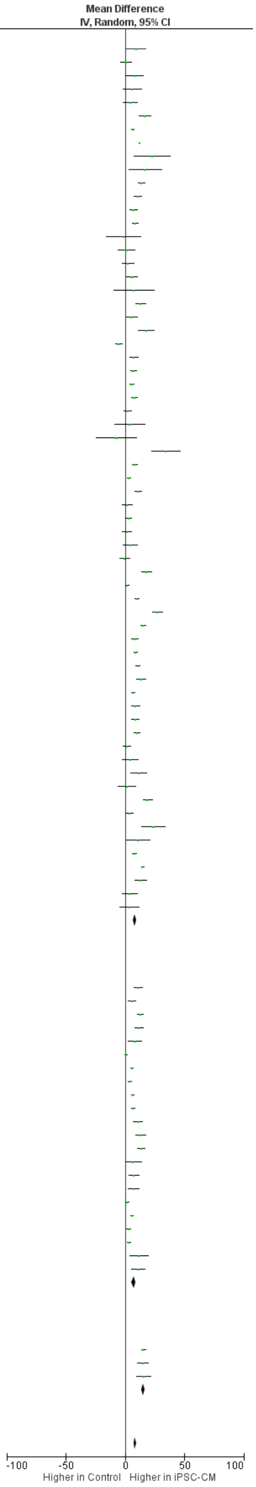

Figure S4. Cell-based effect on animal ejection fraction by time of injection

Study or Subgroup	iPSC-CM Mean	iPSC-CM SD	iPSC-CM Total	Control Mean	Control SD	Control Total	Weight	Mean Difference IV, Random, 95% CI
**10.1.1 Permanent injury**								
A. A. Winters, et al. 2016	39.1	6.1	8	29.7	8.8	6	0.8%	9.40 [1.19, 17.61]
A. A. Winters, et al. 2016	33.4	6.7	17	32.5	6.4	15	1.1%	0.90 [-3.64, 5.44]
A.Chow, et al. 2017	55.3	8	7	47.3	6.1	7	0.8%	8.00 [0.55, 15.45]
A.Chow, et al. 2017	50.6	8.9	9	44.5	7.8	8	0.8%	6.10 [-1.84, 14.04]
A.Tachibana, et al 2017	28.4	9.2	14	24.1	6	10	1.0%	4.30 [-1.79, 10.39]
A.Tachibana, et al 2017	36.6	5.8	14	19.9	5.7	10	1.1%	16.70 [12.04, 21.36]
C.Fan, et als 2019	15.3	1.4	12	8.9	1.4	12	1.4%	6.40 [5.28, 7.52]
C.Fan, et als 2020	25.8	0.4	10	13.6	0.6	10	1.4%	12.20 [11.75, 12.65]
D. Biagi, et als 2021	46.3	3.4	7	35.2	2.9	6	1.2%	11.10 [7.68, 14.52]
F.Zhang, et als 2020	61.77	9.06	6	38.84	16.78	6	0.4%	22.93 [7.67, 38.19]
F.Zhang, et als 2020	62.9	2.6	6	46.1	17.1	6	0.4%	16.80 [2.96, 30.64]
H.Li, et al 2021	34.6	3	5	20.8	1.5	5	1.3%	13.80 [10.86, 16.74]
H.Li, et al 2021	27.8	3.1	5	17.2	1.9	5	1.2%	10.60 [7.41, 13.79]
H.Li, et al 2021	31.1	2.4	5	24.2	2.8	5	1.2%	6.90 [3.67, 10.13]
H.Li, et al 2023	25.9	2	5	17.5	2.2	5	1.3%	8.40 [5.79, 11.01]
J.Li, et al 2017	50.63	6.41	12	37.5	5.15	17	1.1%	13.13 [8.75, 17.51]
J.Stępniewski, et al 2020	30.57	3.02	7	25.14	6.84	11	1.1%	5.43 [0.81, 10.05]
J.Stępniewski, et al 2020	39.02	6.64	7	21.32	7.65	11	0.9%	17.70 [11.02, 24.38]
J.Stępniewski, et al 2020	18.51	3.21	7	23.94	3.42	12	1.3%	-5.43 [-8.50, -2.36]
J.Stępniewski, et al 2020	30.78	3.82	7	23.53	3.42	12	1.2%	7.25 [3.82, 10.68]
J.Yokoyama, et al 2021	50.6	3.7	10	44.8	3.4	10	1.3%	5.80 [2.69, 8.91]
J.Yokoyama, et al 2021	53.7	3.3	10	41	2.3	10	1.3%	12.70 [10.21, 15.19]
K.Miki, et al 2012	48.6	4.7	15	36.9	5	15	1.2%	11.70 [8.23, 15.17]
K.Neef, et al 2021	46.3	4	8	44.1	2.3	9	1.3%	2.20 [-0.95, 5.35]
K.Neef, et al 2021	50	3.3	8	43.1	1.2	9	1.3%	6.90 [4.48, 9.32]
K.Neef, et al 2021	49.5	1.2	8	44	2.5	9	1.3%	5.50 [3.67, 7.33]
K.Neef, et al 2021	51.8	2.8	8	44.2	2.6	9	1.3%	7.60 [5.02, 10.18]
KPMM.Ja, et al 2016	36.68	2.04	4	32.97	18.35	8	0.5%	3.71 [-9.16, 16.58]
KPMM.Ja, et al 2016	25.69	11.35	4	32.97	18.35	8	0.3%	-7.28 [-24.17, 9.61]
L.Citro, et al 2014	81.1	10.5	5	46.9	8.8	5	0.5%	34.20 [22.19, 46.21]
L.Saludas, et al 2019	41.25	2.57	8	33.21	1.86	9	1.3%	8.04 [5.88, 10.20]
L.Saludas, et al 2019	32.7	2.4	11	29.5	1.1	13	1.4%	3.20 [1.66, 4.74]
L.Zhang, et al 2016	43.5	2.1	7	32.5	3.2	7	1.3%	11.00 [8.16, 13.84]
L.Zhang, et al 2016	41.4	2.6	7	39.9	5.1	7	1.2%	1.50 [-2.74, 5.74]
M.Ishida, et al 2019	45.5	2	7	30.2	1.4	8	1.3%	15.30 [13.53, 17.07]
M.Kawamura, et al 2012	53.2	4.3	6	38.3	4.3	6	1.1%	14.90 [10.03, 19.77]
M.Kawamura, et al 2012	51.6	4.9	6	36	5.9	6	1.0%	15.60 [9.46, 21.74]
O.I.García, et al 2015	34.7	5.5	8	33.2	2.3	8	1.2%	1.50 [-2.63, 5.63]
O.I.García, et al 2015	32.6	3.9	8	28.4	8.2	8	1.0%	4.20 [-2.09, 10.49]
O.I.García, et al 2015	35.5	4.7	8	36	4.3	8	1.1%	-0.50 [-4.91, 3.91]
R.Ma, et al 2018	46.43	8.4	16	28.3	1.5	8	1.2%	18.13 [13.88, 22.38]
S.G.Ong, et al 2015	20.8	1.3	8	19.2	1.5	8	1.4%	1.60 [0.22, 2.98]
S.G.Ong, et al 2015	24.4	1.1	8	14.5	1.8	8	1.4%	9.90 [8.44, 11.36]
S.J.Sun, et al 2021	46.3	5.8	8	38	5.5	8	1.0%	8.30 [2.76, 13.84]
S.Mattapally, et al 2018	55.4	5	6	28.3	1.3	6	1.2%	27.10 [22.97, 31.23]
S.V.Rojas, et al 2016	34	4	21	19	2	10	1.3%	15.00 [12.89, 17.11]
S.V.Rojas, et al 2016	47	3	28	39	5	15	1.3%	8.00 [5.24, 10.76]
T.Higuchi, et al 2015	51.09	0.81	5	50.46	0.75	6	1.4%	0.63 [-0.30, 1.56]
T.Higuchi, et al 2015	54	0.7	6	48.1	1.2	6	1.4%	5.90 [4.79, 7.01]
T.Higuchi, et al 2015	53.28	1.2	6	49.52	1	6	1.4%	3.76 [2.51, 5.01]
T.Higuchi, et al 2015	54.6	1	6	48.2	1.1	6	1.4%	6.40 [5.21, 7.59]
T.Higuchi, et al 2015	54.9	0.7	6	48.2	1.3	6	1.4%	6.70 [5.52, 7.88]
T.Samura, et al 2020	44.7	2.5	10	35.8	0.6	10	1.4%	8.90 [7.31, 10.49]
T.Samura, et al 2020	46.7	2.3	10	36.1	0.6	10	1.4%	10.60 [9.13, 12.07]
T.Samura, et al 2020	48.1	5.9	10	34.8	2.5	10	1.2%	13.30 [9.33, 17.27]
T.Samura, et al 2020	42.4	2.5	10	35.8	0.7	10	1.4%	6.60 [4.99, 8.21]
W.Bian, et al 2021	27.6	2.7	6	18.9	3.8	6	1.2%	8.70 [4.97, 12.43]
W.H.Lee, et al 2017	54	3.3	8	45.4	2.8	7	1.3%	8.60 [5.51, 11.69]
W.Zhu, et al 2018	42.2	3.6	9	32.3	2	9	1.3%	9.90 [7.21, 12.59]
W.Zhu, et al 2018	38.4	4.3	10	36.9	2.5	10	1.3%	1.50 [-1.58, 4.58]
X.Guan, et al 2020	52.41	3	9	41.67	5.1	8	1.2%	10.74 [6.70, 14.78]
X.Jiang, et al 2020	50.4	7.6	9	45.9	7	9	0.9%	4.50 [-2.25, 11.25]
X.Jiang, et al 2020	54.7	6.42	9	43.22	7.71	8	0.9%	11.48 [4.69, 18.27]
X.Jiang, et al 2020	48.3	8.1	9	46.73	8.1	9	0.8%	1.57 [-5.91, 9.05]
X.Lou, et al 2019	38.6	5.3	9	19.7	2.2	7	1.2%	18.90 [15.07, 22.73]
X.Lou, et al 2019	30.7	2.6	9	27.2	3.9	7	1.2%	3.50 [0.15, 6.85]
X.Qu, et al 2023	45.3	2.3	7	32.3	5.6	7	1.1%	13.00 [8.52, 17.48]
X.Qu, et al 2023	45.91	3.93	7	32.3	1.9	7	1.2%	13.61 [10.38, 16.84]
X.Qu, et al 2023	43.14	3.48	7	36.3	8.5	7	0.9%	6.84 [0.04, 13.64]
X.Qu, et al 2023	41.84	1.56	7	34.4	5.6	7	1.1%	7.44 [3.13, 11.75]
X.Sun, et al 2020	31.5	5	8	24.5	2.7	4	1.1%	7.00 [2.64, 11.36]
Y.Gao, et al 2021	57.3	6.8	5	33.4	8.8	5	0.7%	23.90 [14.15, 33.65]
Y.Gao, et al 2021	53	6.5	5	42	9.5	5	0.6%	11.00 [0.91, 21.09]
Y.Jiang, et al 2021	56.36	6.18	18	43.22	6.5	9	1.1%	13.14 [8.02, 18.26]
Y.Jiang, et al 2023	39.47	1.75	13	37.85	1.89	13	1.4%	1.62 [0.22, 3.02]
Y.Jiang, et al 2023	39.8	1.2	13	33.9	1.5	13	1.4%	5.90 [4.86, 6.94]
Y.Jiang, et al 2023	39.19	1.61	6	36.17	1.4	6	1.4%	3.02 [1.31, 4.73]
Y.Jiang, et al 2023	39.61	1.26	6	36.45	1.54	6	1.4%	3.16 [1.57, 4.75]
Z.Tao, et al 2021	46.35	10.3	11	42.49	3.86	9	0.9%	3.86 [-2.73, 10.45]
Z.Tao, et al 2021	49.36	10.3	11	45.92	7.73	9	0.8%	3.44 [-4.47, 11.35]
**Subtotal (95% CI)**			**701**			**671**	**90.4%**	**8.37 [7.24, 9.50]**
Heterogeneity: $Tau^2 = 21.46$; $Chi^2 = 1534.58$, df = 79 ($P < 0.00001$); $I^2 = 95\%$								
Test for overall effect: Z = 14.50 ($P < 0.00001$)								
**10.1.2 Ischemic-reperfusion**								
H.m.Li 2023	53.47	7.8	14	54.86	12.08	3	0.4%	-1.39 [-15.66, 12.88]
H.m.Li 2023	50.99	8.99	14	49.79	4.91	3	0.9%	1.20 [-6.08, 8.48]
H.m.Li 2023	45.36	7.76	14	42.82	2.54	3	1.1%	2.54 [-2.44, 7.52]
H.m.Li 2023	51.28	4.6	11	45.77	3.38	3	1.1%	5.51 [0.82, 10.20]
H.m.Li 2023	49.79	7.07	11	42.39	14.36	3	0.3%	7.40 [-9.38, 24.18]
M.Zhao, et al 2021	44.98	2.1	7	42.15	2.77	7	1.3%	2.83 [0.25, 5.41]
Y.H.Fang, et al 2020	56.2	1.1	6	48.6	2	6	1.3%	7.60 [5.77, 9.43]
Y.H.Fang, et al 2020	65.6	1.1	6	51	0.7	6	1.4%	14.60 [13.56, 15.64]
Y.Shiba, et al 2016	57.44	7.3	5	45.94	5.06	5	0.8%	11.50 [3.71, 19.29]
Y.Shiba, et al 2016	60.08	4.21	5	48.88	4.98	5	1.0%	11.20 [5.48, 16.92]
**Subtotal (95% CI)**			**93**			**44**	**9.6%**	**6.78 [2.65, 10.91]**
Heterogeneity: $Tau^2 = 33.24$; $Chi^2 = 124.00$, df = 9 ($P < 0.00001$); $I^2 = 93\%$								
Test for overall effect: Z = 3.22 (P = 0.001)								
**Total (95% CI)**			**794**			**715**	**100.0%**	**8.23 [7.15, 9.32]**
Heterogeneity: $Tau^2 = 21.98$; $Chi^2 = 1715.32$, df = 89 ($P < 0.00001$); $I^2 = 95\%$								
Test for overall effect: Z = 14.85 ($P < 0.00001$)								
Test for subgroup differences: $Chi^2 = 0.53$, df = 1 (P = 0.47), $I^2 = 0\%$								

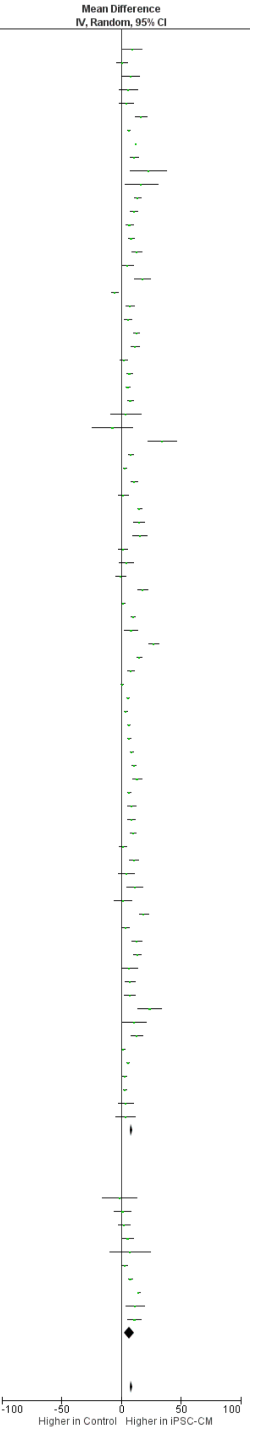

Figure S5. Cell-based effect on animal ejection fraction by disease model

Study or Subgroup	iPSC-CM Mean	iPSC-CM SD	iPSC-CM Total	Control Mean	Control SD	Control Total	Weight	Mean Difference IV, Random, 95% CI
**11.1.1 Xenogeneic**								
A. A. Winters, et al. 2016	39.1	6.1	8	29.7	8.8	6	0.8%	9.40 [1.19, 17.61]
A. A. Winters, et al. 2016	33.4	6.7	17	32.5	6.4	15	1.1%	0.90 [-3.64, 5.44]
A.Chow, et al. 2017	55.3	8	7	47.3	6.1	7	0.8%	8.00 [0.55, 15.45]
A.Chow, et al. 2017	50.6	8.9	9	44.5	7.8	8	0.8%	6.10 [-1.84, 14.04]
A.Tachibana, et al 2017	28.4	9.2	14	24.1	6	10	1.0%	4.30 [-1.79, 10.39]
A.Tachibana, et al 2017	36.6	5.8	14	19.9	5.7	10	1.1%	16.70 [12.04, 21.36]
C.Fan, et als 2019	15.3	1.4	12	8.9	1.4	12	1.4%	6.40 [5.28, 7.52]
C.Fan, et als 2020	25.8	0.4	10	13.6	0.6	10	1.4%	12.20 [11.75, 12.65]
D. Biagi, et als 2021	46.3	3.4	7	35.2	2.9	6	1.2%	11.10 [7.68, 14.52]
F.Zhang, et als 2020	61.77	9.06	6	38.84	16.78	6	0.4%	22.93 [7.67, 38.19]
F.Zhang, et als 2020	62.9	2.6	6	46.1	17.1	6	0.4%	16.80 [2.96, 30.64]
H.Li, et al 2021	34.6	3	5	20.8	1.5	5	1.3%	13.80 [10.86, 16.74]
H.Li, et al 2021	27.8	3.1	5	17.2	1.9	5	1.2%	10.60 [7.41, 13.79]
H.Li, et al 2021	31.1	2.4	5	24.2	2.8	5	1.2%	6.90 [3.67, 10.13]
H.Li, et al 2023	25.9	2	5	17.5	2.2	5	1.3%	8.40 [5.79, 11.01]
H.m.Li 2023	53.47	7.8	14	54.86	12.08	3	0.4%	-1.39 [-15.66, 12.88]
H.m.Li 2023	50.99	8.99	14	49.79	4.91	3	0.9%	1.20 [-6.08, 8.48]
H.m.Li 2023	45.36	7.76	14	42.82	2.54	3	1.1%	2.54 [-2.44, 7.52]
H.m.Li 2023	51.28	4.6	11	45.77	3.38	3	1.1%	5.51 [0.82, 10.20]
H.m.Li 2023	49.79	7.07	11	42.39	14.36	3	0.3%	7.40 [-9.38, 24.18]
J.Li, et al 2017	50.63	6.41	12	37.5	5.15	17	1.1%	13.13 [8.75, 17.51]
J.Stępniewski, et al 2020	30.57	3.02	7	25.14	6.84	11	1.1%	5.43 [0.81, 10.05]
J.Stępniewski, et al 2020	39.02	6.64	7	21.32	7.65	11	0.9%	17.70 [11.02, 24.38]
J.Stępniewski, et al 2020	18.51	3.21	7	23.94	3.42	12	1.3%	-5.43 [-8.50, -2.36]
J.Stępniewski, et al 2020	30.78	3.82	7	23.53	3.42	12	1.2%	7.25 [3.82, 10.68]
J.Yokoyama, et al 2021	50.6	3.7	10	44.8	3.4	10	1.3%	5.80 [2.69, 8.91]
J.Yokoyama, et al 2021	53.7	3.3	10	41	2.3	10	1.3%	12.70 [10.21, 15.19]
KPMM.Ja, et al 2016	36.68	2.04	4	32.97	18.35	8	0.5%	3.71 [-9.16, 16.58]
KPMM.Ja, et al 2016	25.69	11.35	4	32.97	18.35	8	0.3%	-7.28 [-24.17, 9.61]
L.Citro, et al 2014	81.1	10.5	5	46.9	8.8	5	0.5%	34.20 [22.19, 46.21]
L.Saludas, et al 2019	41.25	2.57	8	33.21	1.86	9	1.3%	8.04 [5.88, 10.20]
L.Saludas, et al 2019	32.7	2.4	11	29.5	1.1	13	1.4%	3.20 [1.66, 4.74]
L.Zhang, et al 2016	43.5	2.1	7	32.5	3.2	7	1.3%	11.00 [8.16, 13.84]
L.Zhang, et al 2016	41.4	2.6	7	39.9	5.1	7	1.2%	1.50 [-2.74, 5.74]
M.Ishida, et al 2019	45.5	2	7	30.2	1.4	8	1.3%	15.30 [13.53, 17.07]
M.Kawamura, et al 2012	53.2	4.3	6	38.3	4.3	6	1.1%	14.90 [10.03, 19.77]
M.Kawamura, et al 2012	51.6	4.9	6	36	5.9	6	1.0%	15.60 [9.46, 21.74]
M.Zhao, et al 2021	44.98	2.1	7	42.15	2.77	7	1.3%	2.83 [0.25, 5.41]
R.Ma, et al 2018	46.43	8.4	16	28.3	1.5	8	1.2%	18.13 [13.88, 22.38]
S.G.Ong, et al 2015	24.4	1.1	8	14.5	1.8	8	1.4%	9.90 [8.44, 11.36]
S.G.Ong, et al 2015	20.8	1.3	8	19.2	1.5	8	1.4%	1.60 [0.22, 2.98]
S.J.Sun, et al 2021	46.3	5.8	8	38	5.5	8	1.0%	8.30 [2.76, 13.84]
S.Mattapally, et al 2018	55.4	5	6	28.3	1.3	6	1.2%	27.10 [22.97, 31.23]
T.Samura, et al 2020	44.7	2.5	10	35.8	0.6	10	1.4%	8.90 [7.31, 10.49]
T.Samura, et al 2020	46.7	2.3	10	36.1	0.6	10	1.4%	10.60 [9.13, 12.07]
T.Samura, et al 2020	48.1	5.9	10	34.8	2.5	10	1.2%	13.30 [9.33, 17.27]
T.Samura, et al 2020	42.4	2.5	10	35.8	0.7	10	1.4%	6.60 [4.99, 8.21]
W.Bian, et al 2021	27.6	2.7	6	18.9	3.8	6	1.2%	8.70 [4.97, 12.43]
W.H.Lee, et al 2017	54	3.3	8	45.4	2.8	7	1.3%	8.60 [5.51, 11.69]
W.Zhu, et al 2018	42.2	3.6	9	32.3	2	9	1.3%	9.90 [7.21, 12.59]
W.Zhu, et al 2018	38.4	4.3	10	36.9	2.5	10	1.3%	1.50 [-1.58, 4.58]
X.Guan, et al 2020	52.41	3	9	41.67	5.1	8	1.2%	10.74 [6.70, 14.78]
X.Jiang, et al 2020	50.4	7.6	9	45.9	7	9	0.9%	4.50 [-2.25, 11.25]
X.Jiang, et al 2020	54.7	6.42	9	43.22	7.71	8	0.9%	11.48 [4.69, 18.27]
X.Jiang, et al 2020	48.3	8.1	9	46.73	8.1	9	0.8%	1.57 [-5.91, 9.05]
X.Lou, et al 2019	38.6	5.3	9	19.7	2.2	7	1.2%	18.90 [15.07, 22.73]
X.Lou, et al 2019	30.7	2.6	9	27.2	3.9	7	1.2%	3.50 [0.15, 6.85]
X.Qu, et al 2023	45.3	2.3	7	32.3	5.6	7	1.1%	13.00 [8.52, 17.48]
X.Qu, et al 2023	45.91	3.93	7	32.3	1.9	7	1.2%	13.61 [10.38, 16.84]
X.Qu, et al 2023	43.14	3.48	7	36.3	8.5	7	0.9%	6.84 [0.04, 13.64]
X.Qu, et al 2023	41.84	1.56	7	34.4	5.6	7	1.1%	7.44 [3.13, 11.75]
X.Sun, et al 2020	31.5	5	8	24.5	2.7	4	1.1%	7.00 [2.64, 11.36]
Y.Gao, et al 2021	57.3	6.8	5	33.4	8.8	5	0.7%	23.90 [14.15, 33.65]
Y.Gao, et al 2021	53	6.5	5	42	9.5	5	0.6%	11.00 [0.91, 21.09]
Y.H.Fang, et al 2020	56.2	1.1	6	48.6	2	6	1.3%	7.60 [5.77, 9.43]
Y.H.Fang, et al 2020	65.6	1.1	6	51	0.7	6	1.4%	14.60 [13.56, 15.64]
Y.Jiang, et al 2021	56.36	6.18	18	43.22	6.5	9	1.1%	13.14 [8.02, 18.26]
Y.Jiang, et al 2023	39.47	1.75	13	37.85	1.89	13	1.4%	1.62 [0.22, 3.02]
Y.Jiang, et al 2023	39.8	1.2	13	33.9	1.5	13	1.4%	5.90 [4.86, 6.94]
Y.Jiang, et al 2023	39.19	1.61	6	36.17	1.4	6	1.4%	3.02 [1.31, 4.73]
Y.Jiang, et al 2023	39.61	1.26	6	36.45	1.54	6	1.4%	3.16 [1.57, 4.75]
Z.Tao, et al 2021	46.35	10.3	11	42.49	3.86	9	0.9%	3.86 [-2.73, 10.45]
Z.Tao, et al 2021	49.36	10.3	11	45.92	7.73	9	0.8%	3.44 [-4.47, 11.35]
**Subtotal (95% CI)**			**635**			**575**	**79.0%**	**8.75 [7.53, 9.98]**
Heterogeneity: Tau² = 22.11; Chi² = 1225.18, df = 72 (P < 0.00001); I² = 94%								
Test for overall effect: Z = 14.00 (P < 0.00001)								
**11.1.2 Allogenic**								
K.Miki, et al 2012	48.6	4.7	15	36.9	5	15	1.2%	11.70 [8.23, 15.17]
K.Neef, et al 2021	50	3.3	8	43.1	1.2	9	1.3%	6.90 [4.48, 9.32]
K.Neef, et al 2021	49.5	1.2	8	44	2.5	9	1.3%	5.50 [3.67, 7.33]
K.Neef, et al 2021	51.8	2.8	8	44.2	2.6	9	1.3%	7.60 [5.02, 10.18]
K.Neef, et al 2021	46.3	4	8	44.1	2.3	9	1.3%	2.20 [-0.95, 5.35]
O.I.García, et al 2015	34.7	5.5	8	33.2	2.3	8	1.2%	1.50 [-2.63, 5.63]
O.I.García, et al 2015	32.6	3.9	8	28.4	8.2	8	1.0%	4.20 [-2.09, 10.49]
O.I.García, et al 2015	35.5	4.7	8	36	4.3	8	1.1%	-0.50 [-4.91, 3.91]
S.V.Rojas, et al 2016	34	4	21	19	2	10	1.3%	15.00 [12.89, 17.11]
S.V.Rojas, et al 2016	47	3	28	39	5	15	1.3%	8.00 [5.24, 10.76]
T.Higuchi, et al 2015	51.09	0.81	5	50.46	0.75	6	1.4%	0.63 [-0.30, 1.56]
T.Higuchi, et al 2015	54	0.7	6	48.1	1.2	6	1.4%	5.90 [4.79, 7.01]
T.Higuchi, et al 2015	53.28	1.2	6	49.52	1	6	1.4%	3.76 [2.51, 5.01]
T.Higuchi, et al 2015	54.6	1	6	48.2	1.1	6	1.4%	6.40 [5.21, 7.59]
T.Higuchi, et al 2015	54.9	0.7	6	48.2	1.3	6	1.4%	6.70 [5.52, 7.88]
Y.Shiba, et al 2016	57.44	7.3	5	45.94	5.06	5	0.8%	11.50 [3.71, 19.29]
Y.Shiba, et al 2016	60.08	4.21	5	48.88	4.98	5	1.0%	11.20 [5.48, 16.92]
**Subtotal (95% CI)**			**159**			**140**	**21.0%**	**6.23 [4.40, 8.06]**
Heterogeneity: Tau² = 12.13; Chi² = 234.63, df = 16 (P < 0.00001); I² = 93%								
Test for overall effect: Z = 6.68 (P < 0.00001)								
**Total (95% CI)**			**794**			**715**	**100.0%**	**8.23 [7.15, 9.32]**
Heterogeneity: Tau² = 21.98; Chi² = 1715.32, df = 89 (P < 0.00001); I² = 95%								
Test for overall effect: Z = 14.85 (P < 0.00001)								
Test for subgroup differences: Chi² = 5.06, df = 1 (P = 0.02), I² = 80.2%								

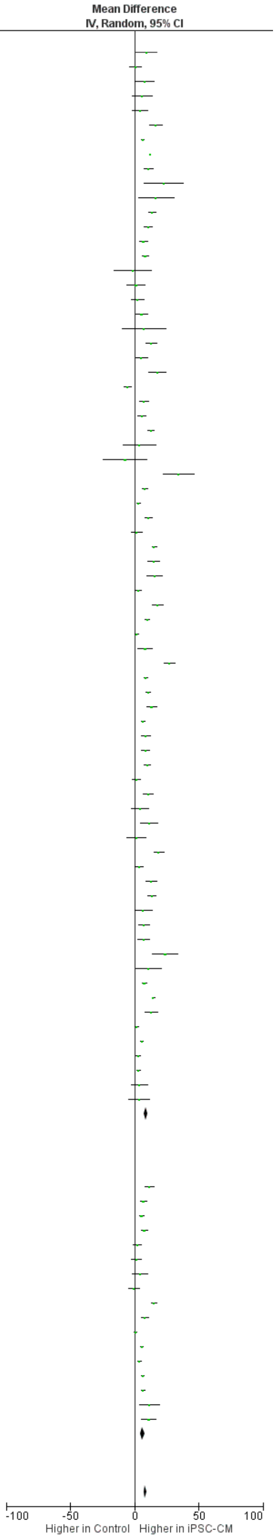

Figure S6. Cell-based effect on animal ejection fraction by origin of cell

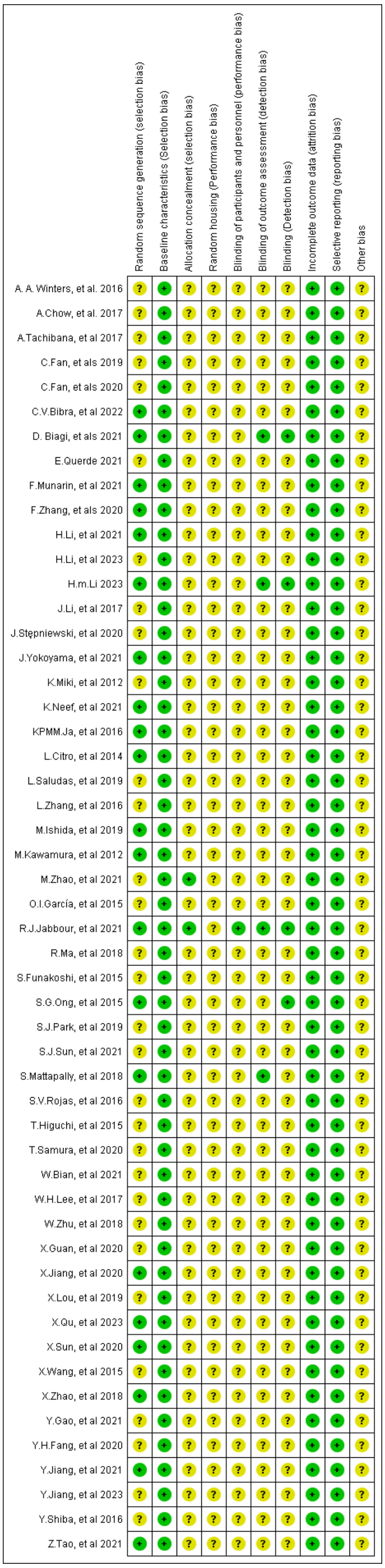

Figure S7. SYRCLE risk of bias. Yellow is unclear risk, green is low risk

Table S1. General information of included studies

Author (year)	Animal characteristics				Intervention characteristics				Outcome measures	
	Species	Age	Method of IHD induced	Immunosuppression	Type of treatment	Total dose	Time of treatment (after IHD-induced)	Control treatment	Time of assessment	Method of cardiac assessment
**Small animal research**										
Atsushi [1] (2017)	SCID mice	90-120 days	permanent LAD ligation	Genetic immunodeficient animal	intramyocardial cell injections	500000	immediately	PBS	2 weeks and 4 weeks	MRI
Amalia [2] (2016)	SCID mice	12 weeks	permanent LAD ligation	Genetic immunodeficient animal	intramyocardial cell injections	500000	immediately	Saline	4 weeks, 12 weeks	Echo
Chow [3] (2017)	nut rat	8 weeks	permanent LAD ligation	NR	intramyocardial cell injections	500000	10 minutes after	PBS	48 hours, 10 weeks	MRI
Diogo [4] (2021)	Wistar rats	8 weeks	permanent LAD ligation	Cyclosporine	intramyocardial cell injections	10000000	1 week after	free solution	30 days	Echo
Fan [5] (2019)	NOD/SCID Gamma mice	NR	permanent LAD ligation	Genetic immunodeficient animal	intramyocardial cell injections	300000	immediately	PBS	6 months	Echo
Fan [6] (2020)	mice	NR	permanent LAD ligation	Genetic immunodeficient animal	bio-engineered tissue treatment	1000000	immediately	free patch	4 weeks	Echo
Fabiola [7] (2021)	Sprague Dawley rat	8-12 weeks	I/R	athymic	bio-engineered tissue treatment	10000000	4 days after	no treatment	1 week, 4 weeks	Echo
Fengzhi [8] (2021)	Sprague–Dawley rats	NR	permanent LAD ligation	NR	intramyocardial cell injections	8000000	10 minutes after	PBS	3 days, 4 weeks	Echo
Hekai [9] (2021)	C57BL/6 mice	6-8 weeks	permanent LAD ligation	Genetic immunodeficient animal	intramyocardial cell injections	200000	immediately	PBS	1 week, 2 weeks, 4 weeks	Echo

Hekai [10] (2023)	C57BL/6 mice	6-8 weeks	permanent LAD ligation	Genetic immunodeficient animal	intramyocardial cell injections	200000	immediately	PBS	4 weeks	Echo
Jacek [11] (2020)	Foxn1nu mice	6-8 weeks	permanent LAD ligation	athymic	intramyocardial cell injections	500000	immediately	Saline	1 week, 2 weeks, 4 weeks, 6 weeks	Echo
Junjun [12] (2017)	nude rats	8 weeks	permanent LAD ligation	no report	bio-engineered tissue treatment	7000000	immediately	free patch	4 weeks	Echo
Junya [13] (2021)	F344/NJcl-rnu/rnu rats	NR	permanent LAD ligation	Genetic immunodeficient animal	bio-engineered tissue treatment	3000000	2 weeks after	free patch	4 weeks, 12 weeks	Echo
Kenji [14] (2012)	F344/NJcl-rnu/rnu rats	NR	permanent LAD ligation	Genetic immunodeficient animal	bio-engineered tissue treatment	NR	2 weeks after	no treatment	1 week, 2 weeks, 4 weeks	Echo
Klaus [15] (2021)	C57BL/6 mice	8-10 weeks	cryo-infarction	Genetic immunodeficient animal	intramyocardial cell injections	500000	immediately	PBS	1 week, 2 weeks, 3 weeks, 4 weeks	MRI
KP My [16] (2015)	SCID mice	10-12 weeks	permanent LAD ligation	Genetic immunodeficient animal	intramyocardial cell injections	200000	immediately	PBS	2 weeks, 4 weeks	Echo
Laura [17] (2019)	BALB/C (Rag-2) mice	8-12 weeks	permanent LAD ligation	Genetic immunodeficient animal	intramyocardial cell injections	150000	15 minutes after	free solution	7 days, 60 days	Echo
Liying [18] (2015)	NOD/SCID/γc−/− (NSG) mice	12 weeks	permanent LAD ligation	Genetic immunodeficient animal	bio-engineered tissue treatment	600000	immediately	free solution	1 week, 4 weeks	Echo
Lucas [19] (2014)	nude rats	NR	permanent LAD ligation	athymic	intramyocardial injections	1000000	immediately	free solution	4 weeks	Echo
Olalla [20] (2015)	DBA/2J mice	8 weeks	permanent coronary ligation	Genetic immunodeficient animal	intramyocardial injections	200000	15 minutes after	PBS	3 days, 60 days	Echo
Ruilian [21] (2018)	SCID mice	10-12 weeks	permanent coronary ligation	Genetic immunodeficient animal	intramyocardial cell injections	200000	immediately	PBS	4 weeks	Echo
Saidulu [22] (2018)	NOD/SCID Gamma mice	NR	permanent LAD ligation	Genetic immunodeficient animal	bio-engineered tissue treatment	200000	immediately	no treatment	4 weeks	Echo

Sang-Ging [23] (2015)	NOD/SCID mice	NR	permanent LAD ligation	Genetic immunodeficient animal	intramyocardial cell injections	2000000	immediately	Saline	7 days, 35 days	MRI
Sebastian [24] (2017)	SCID beige mice	NR	permanent LAD ligation	Genetic immunodeficient animal	intramyocardial cell injections	1500000	immediately	PBS	3 days, 7 days, 14 days, 21 days, 28 days	MRI
Shunsuke [25] (2015)	NOG mice	NR	permanent LAD ligation	Genetic immunodeficient animal	intramyocardial cell injections	1000000	immediately	PBS	1 week, 2 weeks, 3 weeks, 4 weeks, 6 weeks, 8 weeks	Echo
Si-Jia [26] (2021)	ICR mice	12-16 weeks	permanent LAD ligation	Genetic immunodeficient animal	intramyocardial cell injections	1000000	immediately	PBS	4wks	Echo
Soon-Jung [27] (2019)	Fischer 344 rat	8-10 weeks	permanent LAD ligation	Genetic immunodeficient animal	intramyocardial cell injections	1000000	7 days after	no treatment	2 weeks, 4 weeks, 8 weeks	Echo
Takaaki [28] (2020)	nude rat	8 weeks	permanent LAD ligation	athymic	bio-engineered tissue treatment	5000000	immediately	free sheet	1 week, 2 weeks, 3 weeks, 4 weeks	Echo
Takahiro [29] (2015)	F344/NJcl-rnu/rnu rats	6 weeks	permanent LAD ligation	Genetic immunodeficient animal	bio-engineered tissue treatment	NR	2 weeks after	no treatment	7 days, 14 days	Echo
Weihua [30] (2021)	NOD/SCID Gamma mice	NR	permanent LAD ligation	Genetic immunodeficient animal	intramyocardial cell injections	300000	immediately	PBS	4 weeks	Echo
Won [31] (2017)	nude rat	NR	permanent LAD ligation	athymic	intramyocardial cell injections	10000000	4 days after	PBS	30 days	MRI
Wuqiang [32] (2018)	NOD/SCID Gamma mice	NR	permanent LAD ligation	Genetic immunodeficient animal	intramyocardial cell injections	300000	immediately	PBS	1 week, 4 weeks	Echo
Xi [33] (2019)	NOD/ SCID gamma mic	8-10w	permanent LAD ligation	Genetic immunodeficient animal	intramyocardial cell injections	300000	immediately	no treatment	1 week, 4 weeks	Echo
Xiang [34] (2023)	NJcl-rnu/rnu nude rat	7w	permanent LAD ligation	Genetic immunodeficient animal	bio-engineered tissue treatment	5000000	2 weeks after	no treatment	1 week, 2 weeks, 3 weeks, 4 weeks	Echo

Xin Jiang [35] (2020)	ICR mice	12-16w	permanent LAD ligation	Genetic immunodeficient animal	intramyocardial cell injections	400000	10 minutes after	free solution	1 week, 2 weeks, 4 weeks	Echo
Xintong [36] (2015)	nude rats	NR	permanent LAD ligation	NR	intramyocardial cell injections	3000000	30 minutes after	PBS	2 weeks	Echo
Xuetao [37] (2020)	rnu/rnu rat	7-8 weeks	permanent LAD ligation	athymic	intramyocardial cell injections	10000000	2 weeks after	no treatment	4 weeks	Echo
Xumin [38] (2020)	Sprague Dawley rat	8 weeks	permanent LAD ligation	methylprednisolone	intramyocardial cell injections	10000000	10 days after	Albumin solution	4 weeks	Echo
Yi-Hsien [39] (2020)	Sprague Dawley rat	12 weeks	temporary LAD ligation	cyclosporine	intramyocardial cell injections	10000000	4 days after	free solution	4 weeks	Echo
Yu Jiang [40] (2021)	Sprague Dawley rat	NR	permanent LAD ligation	NR	1. intramyocardial cell injections 2. bio-engineered tissue treatment	1000000	immediately	Saline	4 weeks	Echo
Yuanxue [41] (2021)	Sprague–Dawley rat	NR	permanent LAD ligation	no report	intramyocardial cell injections	10000000	immediately	PBS	1 week, 5 weeks	Echo
Yun [42] (2023)	C57BL/6J mice	11-12 weeks	permanent LAD ligation	no report	bio-engineered tissue treatment	200000	7 days after	free solution	7 days, 28 days, 60 days, 90 days	Echo
Zhonghao [43] (2020)	Wistar rat	12 weeks	permanent LAD ligation	cyclosporine	bio-engineered tissue treatment	1000000	10 minutes after	free solution	6 weeks	Echo
**Large animal research**										
Constantin [44] (2022)	guinea pigs	NR	cryo-infarction	cyclosporine, methylprednisolone	bio-engineered tissue treatment	15000000	4 weeks after	free patch	4 weeks	Echo
Eva [45] (2021)	guinea pigs	8-9 weeks	cryo-infarction	cyclosporine, methylprednisolone	bio-engineered tissue treatment	9000000, 12000000, 15000000	7 days after	free patch	4ws	Echo

Hong-mei [46] (2023)	primate	4-6y years	I/R	methylprednisolone, mycophenolate mofetil, tacrolimus	1. intravenous, 2. intramyocardial 3. intracoronary cell injections,	100000000/ kg, 100000 (coronary)	24 hours after	no treatment	1 week, 2 weeks, 4 weeks, 6 weeks ,8 weeks, 12 weeks	Echo
Masashi [47] (2011)	minipig	NR	permanent LAD ligation	tacrolimus	bio-engineered tissue treatment	25000000	1 month after	no treatment	4 weeks, 8 weeks	Echo and CT scan
Masaru [48] (2019)	CLAWN miniature porcine	6-10 month s	permanent LAD ligation	tacrolimus, mycophenolate mofetil, corticosteroids	bio-engineered tissue treatment	100000000	1 month after	no treatment	8	Echo and MRI
Meng [49] (2021)	Yorkshire pig	NR	I/R	no report	intramyocardial cell injections	60000000	60 minutes after	PBS	4 weeks	MRI
Richard [50] (2021)	New Zealand white rabbit	NR	permanent coronary ligation	ciclosporin, methylprednisolone	bio-engineered tissue treatment	17500000	immediately	free patch	1 week, 2 weeks, 4 weeks	Echo
Yuji [51] (2016)	Filipino cynomolgus monkeys	4-5 years	I/R	methylprednisolone, tacrolimus	intramyocardial cell injections	400000000	2 weeks after	free solution	4wks, 12wks	Echo

NR: not report

I/R: ischemia/reperfusion therapy

List of included research

1. Tachibana, A., et al., *Paracrine Effects of the Pluripotent Stem Cell-Derived Cardiac Myocytes Salvage the Injured Myocardium.* Circ Res, 2017. **121**(6): p. e22-e36.
2. Winters, A.A., et al., *Evaluation of Multiple Biological Therapies for Ischemic Cardiac Disease.* Cell Transplant, 2016. **25**(9): p. 1591-1607.
3. Chow, A., et al., *Human Induced Pluripotent Stem Cell-Derived Cardiomyocyte Encapsulating Bioactive Hydrogels Improve Rat Heart Function Post Myocardial Infarction.* Stem Cell Reports, 2017. **9**(5): p. 1415-1422.
4. Biagi, D., et al., *In Situ Maturated Early-Stage Human-Induced Pluripotent Stem Cell-Derived Cardiomyocytes Improve Cardiac Function by Enhancing Segmental Contraction in Infarcted Rats.* J Pers Med, 2021. **11**(5).
5. Fan, C., et al., *Cardiomyocytes from CCND2-overexpressing human induced-pluripotent stem cells repopulate the myocardial scar in mice: A 6-month study.* J Mol Cell Cardiol, 2019. **137**: p. 25-33.
6. Fan, C., et al., *CHIR99021 and fibroblast growth factor 1 enhance the regenerative potency of human cardiac muscle patch after myocardial infarction in mice.* J Mol Cell Cardiol, 2020. **141**: p. 1-10.
7. Munarin, F., et al., *Engineered human myocardium with local release of angiogenic proteins improves vascularization and cardiac function in injured rat hearts.* Biomaterials, 2020. **251**: p. 120033.
8. Zhang, F., et al., *Transferrin improved the generation of cardiomyocyte from human pluripotent stem cells for myocardial infarction repair.* J Mol Histol, 2021. **52**(1): p. 87-99.
9. Li, H., et al., *Injectable AuNP-HA matrix with localized stiffness enhances the formation of gap junction in engrafted human induced pluripotent stem cell-derived cardiomyocytes and promotes cardiac repair.* Biomaterials, 2021. **279**: p. 121231.
10. Li, H., et al., *Supramolecular Assemblies of Glycopeptides Enhance Gap Junction Maturation of Human Induced Pluripotent Stem Cell-Derived Cardiomyocytes via Inducing Spheroids Formation to Optimize Cardiac Repair.* Adv Healthc Mater, 2023. **12**(25): p. e2300696.
11. Stępniewski, J., et al., *Human Induced Pluripotent Stem Cell-Derived Cardiomyocytes, in Contrast to Adipose Tissue-Derived Stromal Cells, Efficiently Improve Heart Function in Murine Model of Myocardial Infarction.* Biomedicines, 2020. **8**(12).
12. Li, J., et al., *Human Pluripotent Stem Cell-Derived Cardiac Tissue-like Constructs for Repairing the Infarcted Myocardium.* Stem Cell Reports, 2017. **9**(5): p. 1546-1559.
13. Yokoyama, J., et al., *Human induced pluripotent stem cell-derived three-dimensional cardiomyocyte tissues ameliorate the rat ischemic myocardium by remodeling the extracellular matrix and cardiac protein phenotype.* PLoS One, 2021. **16**(3): p. e0245571.
14. Miki, K., et al., *Bioengineered myocardium derived from induced pluripotent stem cells improves cardiac function and attenuates cardiac remodeling following chronic myocardial infarction in rats.* Stem Cells Transl Med, 2012. **1**(5): p. 430-7.

15. Neef, K., et al., *Co-transplantation of Mesenchymal Stromal Cells and Induced Pluripotent Stem Cell-Derived Cardiomyocytes Improves Cardiac Function After Myocardial Damage.* Front Cardiovasc Med, 2021. **8**: p. 794690.
16. Ja, K.P., et al., *iPSC-derived human cardiac progenitor cells improve ventricular remodelling via angiogenesis and interstitial networking of infarcted myocardium.* J Cell Mol Med, 2016. **20**(2): p. 323-32.
17. Saludas, L., et al., *Long-Term Engraftment of Human Cardiomyocytes Combined with Biodegradable Microparticles Induces Heart Repair.* J Pharmacol Exp Ther, 2019. **370**(3): p. 761-771.
18. Zhang, L., et al., *Derivation and high engraftment of patient-specific cardiomyocyte sheet using induced pluripotent stem cells generated from adult cardiac fibroblast.* Circ Heart Fail, 2015. **8**(1): p. 156-66.
19. Citro, L., et al., *Comparison of human induced pluripotent stem-cell derived cardiomyocytes with human mesenchymal stem cells following acute myocardial infarction.* PLoS One, 2014. **9**(12): p. e116281.
20. Iglesias-García, O., et al., *Neuregulin-1β induces mature ventricular cardiac differentiation from induced pluripotent stem cells contributing to cardiac tissue repair.* Stem Cells Dev, 2015. **24**(4): p. 484-96.
21. Ma, R., et al., *Electrical Stimulation Enhances Cardiac Differentiation of Human Induced Pluripotent Stem Cells for Myocardial Infarction Therapy.* Antioxid Redox Signal, 2018. **28**(5): p. 371-384.
22. Mattapally, S., et al., *Spheroids of cardiomyocytes derived from human-induced pluripotent stem cells improve recovery from myocardial injury in mice.* Am J Physiol Heart Circ Physiol, 2018. **315**(2): p. H327-h339.
23. Ong, S.G., et al., *Microfluidic Single-Cell Analysis of Transplanted Human Induced Pluripotent Stem Cell-Derived Cardiomyocytes After Acute Myocardial Infarction.* Circulation, 2015. **132**(8): p. 762-771.
24. Rojas, S.V., et al., *Transplantation of purified iPSC-derived cardiomyocytes in myocardial infarction.* PLoS One, 2017. **12**(5): p. e0173222.
25. Funakoshi, S., et al., *Enhanced engraftment, proliferation and therapeutic potential in heart using optimized human iPSC-derived cardiomyocytes.* Scientific Reports, 2016. **6**(1): p. 19111.
26. Sun, S.J., et al., *Immunomodulation by systemic administration of human-induced pluripotent stem cell-derived mesenchymal stromal cells to enhance the therapeutic efficacy of cell-based therapy for treatment of myocardial infarction.* Theranostics, 2021. **11**(4): p. 1641-1654.
27. Park, S.-J., et al., *Dual stem cell therapy synergistically improves cardiac function and vascular regeneration following myocardial infarction.* Nature Communications, 2019. **10**(1): p. 3123.
28. Samura, T., et al., *Laminin-221 Enhances Therapeutic Effects of Human-Induced Pluripotent Stem Cell-Derived 3-Dimensional Engineered Cardiac Tissue Transplantation in a Rat Ischemic Cardiomyopathy Model.* J Am Heart Assoc, 2020. **9**(16): p. e015841.
29. Higuchi, T., et al., *Functional and Electrical Integration of Induced Pluripotent Stem Cell-Derived Cardiomyocytes in a Myocardial Infarction Rat Heart.* Cell Transplant, 2015. **24**(12): p. 2479-89.

30. Bian, W., et al., *miR-199a Overexpression Enhances the Potency of Human Induced-Pluripotent Stem-Cell-Derived Cardiomyocytes for Myocardial Repair.* Front Pharmacol, 2021. **12**: p. 673621.
31. Lee, W.H., et al., *Comparison of Non-Coding RNAs in Exosomes and Functional Efficacy of Human Embryonic Stem Cell- versus Induced Pluripotent Stem Cell-Derived Cardiomyocytes.* Stem Cells, 2017. **35**(10): p. 2138-2149.
32. Zhu, W., et al., *CCND2 Overexpression Enhances the Regenerative Potency of Human Induced Pluripotent Stem Cell-Derived Cardiomyocytes: Remuscularization of Injured Ventricle.* Circ Res, 2018. **122**(1): p. 88-96.
33. Lou, X., et al., *N-cadherin overexpression enhances the reparative potency of human-induced pluripotent stem cell-derived cardiac myocytes in infarcted mouse hearts.* Cardiovasc Res, 2020. **116**(3): p. 671-685.
34. Qu, X., et al., *ONO-1301 enhances post-transplantation survival of human induced pluripotent stem cell-derived cardiac tissue sheet by promoting angiogenesis.* J Heart Lung Transplant, 2023. **42**(6): p. 716-729.
35. Jiang, X., Z. Yang, and M. Dong, *Cardiac repair in a murine model of myocardial infarction with human induced pluripotent stem cell-derived cardiomyocytes.* Stem Cell Res Ther, 2020. **11**(1): p. 297.
36. Wang, X., et al., *A temperature-sensitive, self-adhesive hydrogel to deliver iPSC-derived cardiomyocytes for heart repair.* Int J Cardiol, 2015. **190**: p. 177-80.
37. Sun, X., et al., *Transplanted microvessels improve pluripotent stem cell-derived cardiomyocyte engraftment and cardiac function after infarction in rats.* Sci Transl Med, 2020. **12**(562).
38. Guan, X., et al., *Transplantation of human induced pluripotent stem cell-derived cardiomyocytes improves myocardial function and reverses ventricular remodeling in infarcted rat hearts.* Stem Cell Res Ther, 2020. **11**(1): p. 73.
39. Fang, Y.H., et al., *Efficient Cardiac Differentiation of Human Amniotic Fluid-Derived Stem Cells into Induced Pluripotent Stem Cells and Their Potential Immune Privilege.* Int J Mol Sci, 2020. **21**(7).
40. Jiang, Y., et al., *Myocardial repair of bioengineered cardiac patches with decellularized placental scaffold and human-induced pluripotent stem cells in a rat model of myocardial infarction.* Stem Cell Res Ther, 2021. **12**(1): p. 13.
41. Gao, Y., et al., *CRISPR/Cas9-edited triple-fusion reporter gene imaging of dynamics and function of transplanted human urinary-induced pluripotent stem cell-derived cardiomyocytes.* Eur J Nucl Med Mol Imaging, 2021. **48**(3): p. 708-720.
42. Jiang, Y., et al., *Dual human iPSC-derived cardiac lineage cell-seeding extracellular matrix patches promote regeneration and long-term repair of infarcted hearts.* Bioact Mater, 2023. **28**: p. 206-226.
43. Tao, Z., et al., *Angiopoietin-1 enhanced myocyte mitosis, engraftment, and the reparability of hiPSC-CMs for treatment of myocardial infarction.* Cardiovasc Res, 2021. **117**(6): p. 1578-1591.
44. von Bibra, C., et al., *Human engineered heart tissue transplantation in a guinea pig chronic injury model.* J Mol Cell Cardiol, 2022. **166**: p. 1-10.
45. Querdel, E., et al., *Human Engineered Heart Tissue Patches Remuscularize the Injured Heart in a Dose-Dependent Manner.* Circulation, 2021. **143**(20): p. 1991-2006.

46. Li, H.M., et al., *Optimal transplantation strategy using human induced pluripotent stem cell-derived cardiomyocytes for acute myocardial infarction in nonhuman primates.* MedComm (2020), 2023. **4**(3): p. e289.
47. Kawamura, M., et al., *Feasibility, safety, and therapeutic efficacy of human induced pluripotent stem cell-derived cardiomyocyte sheets in a porcine ischemic cardiomyopathy model.* Circulation, 2012. **126**(11 Suppl 1): p. S29-37.
48. Ishida, M., et al., *Transplantation of Human-induced Pluripotent Stem Cell-derived Cardiomyocytes Is Superior to Somatic Stem Cell Therapy for Restoring Cardiac Function and Oxygen Consumption in a Porcine Model of Myocardial Infarction.* Transplantation, 2019. **103**(2): p. 291-298.
49. Zhao, M., et al., *Cyclin D2 Overexpression Enhances the Efficacy of Human Induced Pluripotent Stem Cell-Derived Cardiomyocytes for Myocardial Repair in a Swine Model of Myocardial Infarction.* Circulation, 2021. **144**(3): p. 210-228.
50. Jabbour, R.J., et al., *In vivo grafting of large engineered heart tissue patches for cardiac repair.* JCI Insight, 2021. **6**(15).
51. Shiba, Y., et al., *Allogeneic transplantation of iPS cell-derived cardiomyocytes regenerates primate hearts.* Nature, 2016. **538**(7625): p. 388-391.

Table S2. Meta-regression of potential modifiers of FS

Variable	Coefficient	SE	t-value	P-value	95% Confidence Interval
Animal size -small	14.11	3.32	4.25	0.00	7.45 to 20.78
Treatment timing: chronic	13.36	5.45	2.45	0.02	2.44 to 24.28
Treatment timing: subacute	-2.10	1.74	-1.20	0.23	-5.59 to 1.40
Method delivery: Intracoronary	-8.39	3.34	-2.51	0.02	-15.10 to -1.69
Method delivery: Intramyocardial	-0.73	1.51	-0.48	0.63	-3.76 to 2.31
Method delivery: Intravenous	-1.03	2.97	-0.35	0.73	-6.98 to 4.91
Time of follow up: < 4w	-1.87	1.09	-1.72	0.09	-4.06 to 0.31
Time of follow up: > 8w	-0.63	1.89	-0.33	0.74	-4.43 to 3.17
Cell origin: Xenogeneic	-4.63	2.96	-1.57	0.12	-10.57 to 1.30
Disease model: Permanent injury	-7.36	2.92	-2.52	0.01	-13.22 to -1.5

Table S3. Meta-regression of potential modifiers of LV fibrosis

Variable	Coefficient	SE	t-value	P-value	95% Confidence Interval
Animal size: big	-1.64	2.31	-0.71	0.48	-6.31 to 3.01
Animal size: small	3.51	2.91	1.21	0.23	-2.28 to 9.31
Treatment timing: acute	-3.11	2.26	-1.37	0.17	-7.61 to 1.39
Treatment timing: chronic	8.16	4.65	1.75	0.08	-1.08 to 17.41
Treatment timing: sub-acute	-3.19	2.21	-1.44	0.15	-7.59 to 1.21
Method delivery: Bio-engineered tissue	3.91	2.14	1.82	0.07	-0.34 to 8.18
Method delivery: Intracoronary	-8.06	3.32	-2.42	0.**02**	-14.67 to -1.45
Method delivery: Intramyocardial	3.82	1.64	2.33	**0.02**	0.56 to 7.08
Method delivery: Intravenous	2.18	2.81	0.78	0.43	-3.37 to 7.75
Cell origin: Allogenous	-0.15	1.13	-0.13	0.89	-2.41 to 2.11
Cell origin: Xenogeneic	2.01	0.92	2.18	**0.03**	0.18 to 3.84
Disease model: IR	1.96	2.61	0.74	0.45	-3.24 to 7.16
Disease model: Permanent injury	-0.09	2.15	-0.04	0.96	-4.38 to 4.18
Follow up: < 4 weeks	1.01	1.29	0.77	0.43	-1.57 to 3.59
Follow up: 4-8 weeks	1.05	1.26	0.82	0.41	-1.47 to 3.57
Follow up: > 8 weeks	-0.96	2.45	-0.39	0.69	-5.84 to 3.91

Table S4. Meta-regression of potential modifiers of LVESV

Variable	Coefficient	SE	t-value	P-value	95% Confidence Interval
Treatment timing: sub-acute	-100.48	69.60	-1.44	0.169	-248.83 to 47.87
Method delivery: Intramyocardial	34.21	20.06	1.71	0.109	-8.56 to 76.97

Time of follow – up: < 4w	0.99	5.06	0.20	0.847	-9.79 to 11.77
Time of follow – up: 8w	-30.45	10.72	-2.84	0.012	-53.30 to -7.60
Cell origin: Xenogeneic	-5.17	8.22	-0.63	0.539	-22.70 to 12.35

Table S5. Meta-regression of potential modifiers of LVEDV

Variable	Coefficient	SE	t-value	P-Value	95% Confidence Interval
Animal size: small	-13.97	4.47	-3.12	0.005	-23.38 to -4.56
Treatment timing: acute	23.77	10.84	2.19	0.04	0.98 to 46.55
Treatment timing: sub-acute	-37.74	14.31	-2.63	0.01	-67.81 to -7.68
Method delivery: Bio-engineered tissue	-27.47	10.82	-2.53	0.02	-50.21 to -4.72
Method delivery: Intramyocardial	13.49	8.14	1.65	0.1	-3.61 to 30.59
Time of follow – up: 4w-8w	-3.59	6.94	-0.51	0.61	-18.18 to 10.99
Time of follow - up: < 4w	-9.15	7.67	-1.19	0.24	-25.28 to 6.97
Time of follow – up: > 8w	-1.2266	10.3233	-0.11	0.9	-22.91 to 20.46
Cell origin: Allogenous	-4.71	7.11	-0.67	0.51	-19.72 to 10.18
Cell origin: Xenogeneic	-9.20	5.73	-1.61	0.12	-21.25 to 2.85
Disease model: Permanent injury	-13.97	4.47	-3.12	0.006	-23.38 to -4.56
